# DNA Methylation Expression Profile of Blood Heat Syndrome and Blood Stasis Syndrome in TCM Psoriasis

**DOI:** 10.1155/2022/9343285

**Published:** 2022-09-19

**Authors:** Meng Xing, Ying Luo, Le Kuai, Yi Ru, Xiaojie Ding, Xiaoninng Yan, Bin Li, Xin Li

**Affiliations:** ^1^Department of Dermatology, Shaanxi Hospital of Traditional Chinese Medicine, Xi'an 710003, China; ^2^Department of Dermatology, Yueyang Hospital of Integrated Traditional Chinese and Western Medicine, Shanghai University of Traditional Chinese Medicine, Shanghai 200437, China; ^3^Institute of Dermatology, Shanghai Academy of Traditional Chinese Medicine, Shanghai 201203, China; ^4^Shanghai Skin Disease Hospital, School of Medicine, Tongji University, Shanghai 200443, China

## Abstract

**Objective:**

Traditional Chinese medicine (TCM) emphasizes treatment based on syndrome differentiation. This study aimed to clarify the characteristics of DNA methylation expression profiles in peripheral blood mononuclear cells (PBMCs) in patients with psoriasis and analyze the differences in these profiles among different TCM syndromes of psoriasis in order to provide a material basis for the diversity of these syndromes.

**Methods:**

Blood samples were collected from 32 participants, including 14 patients with psoriatic blood heat syndrome (BHS), 12 patients with psoriatic blood stasis syndrome (BSS), and 6 healthy controls. PBMCs were extracted and subjected to DNA quality inspection. An Illumina Human Methylation 850k chip was used to sequence each group of samples. According to gene annotation classification together with CpG island annotation classification, the differentially methylated regions between sample groups were screened, while Gene Ontology and Kyoto Encyclopedia of Genes and Genomes pathway analyses were applied to perform functional analyses of DMGs. Finally, the DMGs closely correlating with psoriatic severity were screened using Spearman's correlation analysis.

**Results:**

Compared with normal controls, patients with psoriasis showed an overall trend of hypermethylation. In psoriasis, the differential methylation probes were mainly distributed on gene body region on the genome, while those in CpG regions were mainly distributed in CpG islands. Compared with healthy controls, the overall trends in methylation were similar in psoriatic BHS and BSS patients compared to healthy controls. However, bioinformatic analysis revealed different functions of DMGs. We also found that the methylation levels of *TRIM14* and *PRDM16* were closely correlated with PASI scores and could serve as potential biomarkers to assess the severity of psoriasis.

**Conclusions:**

Our study, for the first time, indicated the possible involvement of DNA methylation in regulating the characteristics of TCM syndromes of psoriasis, providing a new direction for research into TCM psoriatic syndromes.

## 1. Introduction

Psoriasis is a chronic, immune-mediated skin disease that affects 2-3% of the global population [1]. The pathogenesis of psoriasis is still not fully understood. At present, it is believed to be caused by a disorder of the immune system based on the mutual influence of genetic and environmental factors. Therefore, increasing evidence points to the role of epigenetics, particularly DNA methylation [2–4], which is a covalent modification, selectively catalyzed by a methyltransferase, of the cytosine in the CpG dinucleotide, to form 5-methylcytosine [5]. This type of methylation occurs primarily in CpG islands (CGIs) mainly located in the promoter and exon regions of structural genes; these islands thus often participate in gene transcription regulation [6, 7] as a reversible and heritable epigenetic process.

Traditional Chinese medicine (TCM) is a complementary and alternative therapy that has played a positive role in the treatment of psoriasis [8]. TCM usually adopts a treatment principle based on syndrome differentiation; thus, psoriasis is divided into different syndrome types to adopt different therapeutic regimens. Blood heat syndrome (BHS) and blood stasis syndrome (BSS) are the most common types of psoriasis vulgaris [9]. The syndrome theory of TCM is based on the holistic concept of “man is an integral part of nature”, a pathological summary of the body at a certain stage in the development of the disease, which emphasizes the interaction between time, space, the human body, and the external environment, thus reflecting the overall state of the body at that stage, consistently with the emphasis of epigenetics on the important role of acquired environmental factors in human diseases. TCM syndromes are one of the quintessential tenets of TCM theory, which determined the implementation of clinical therapeutic regimens and clinical efficacy. Currently, TCM syndromes remain within the description of macroscopic clinical phenomena. The practice of dialectics is based on the personal clinical experience of doctors; thus, there is a lack of precise and quantitative systematic scientific evaluation indicators. Based on the similarity in the concepts of epigenetics together with TCM, research ideas and methods for these may overlap. Therefore, the introduction of epigenetics into the study of TCM syndromes will provide new insights into the study of TCM syndromes.

## 2. Materials and Methods

### 2.1. Study Sample

Psoriasis patients (*n* = 26) and healthy subjects (*n* = 6) were recruited from the Dermatology Clinic, Yueyang Hospital of Integrated Traditional Chinese and Western Medicine, Shanghai, China, and written consent was obtained for participation in the study. Basic subjects' information, psoriasis area, and severity index (PASI) [10] were recorded. Sample characteristics are summarized in Table S1. The disease was diagnosed by at least two dermatologists. Patients with generalized psoriasis vulgaris who met the diagnostic criteria (Table S2) of BHS (*n* = 14) and BSS (*n* = 12) for psoriasis according to TCM [11] were enrolled in order to minimize clinical heterogeneity. Patients were denied any systemic or topical therapy for at least 1 month prior to sample collection. Then, 2 mL of blood was drawn into an EDTA tube for each patient, placed in a refrigerator at −20°C, and peripheral blood mononuclear cells (PBMCs) were isolated within 24 h.

### 2.2. DNA Methylation Study and Data Analyses

The Illumina Infinium Human Methylation 850 BeadChip operation manual was strictly followed to complete DNA extraction, transformation, amplification, and sulfite treatment, among others. The QC report test indicated that the quality control indicators of the samples met the Illumina quality requirements and thus further methylation data analyses could be performed (Figure S1). Data analyses were assisted by Shanghai Biochip Company. The original data of the chip were first preprocessed with *R* software (version 3.6.3) minfi package, and then the *R* software IMA package was used to screen for differences in methylation sites and methylation regions between the groups. The analyses process of entire methylation was divided into four steps: data quality control, preprocessing, analyses of DMSs, and analyses of differentially methylated regions. The beta value (*β*) is generally used as an index to measure the degree of methylation of the relevant site. The value interval is (0, 1); a value closer to 1 indicates a higher degree of methylation of the site, while a value closer to 0 indicates a lower degree of methylation of the site. To assess the DMSs between groups, we used the pool *t*-test method using the screening criteria of *p*value < 0.05 and |beta difference| > 0.14.

### 2.3. Gene Ontology (GO) Enrichment Analysis and Kyoto Encyclopedia of Genes and Genomes (KEGG) Pathway Enrichment Analysis

The cluster profiler package in *R* software (version 3.6.3) was used to perform enrichment analyses of GO and KEGG terms, map the selected differentially methylated region- (DMR-) related genes to the terms of the GO and KEGG pathway databases, and calculate the number of genes in each entry. We then applied the hypergeometric test to screen the GO or KEGG entries that were significantly enriched in DMR-related genes as compared with the entire genome background. After the calculated *p* value was corrected by Bonferroni correction, a corrected *p*value (FDR) genome was used as the threshold. GO or KEGG terms that met this condition were considered significantly enriched in differentially expressed genes.

### 2.4. Reverse Transcription-Polymerase Chain Reaction (RT-PCR)

PBMCs were extracted from peripheral blood of healthy subjects as well as psoriasis patients and stored at −20°C. RT-PCR was used to measure the mRNA levels of *TRIM14* and *PRDM16* in PBMCs. Firstly, cDNA is synthesized from RNA by reverse transcriptase, and then cDNA is used as template to amplify and synthesize the target fragment under the action of DNA polymerase. Primers usedwere as follows: *TRIM14* (F: GAGGTGGAGATGAATGGCGG, and R: TGCTGCTGCTTCTTGATTGC) and *PRDM16* (F: TCCGAAGACACTCCTCTCCA, and R: AAATGCTCCAGACTCCGACG).

### 2.5. Enzyme Linked Immunosorbent Assay (ELISA)

The protein levels of TRIM14 and PRDM16 were measured by ELISA. TRIM14 ELISA Kit (EH13190, Wuhan Fine Biotech Co., Ltd.) and PRDM16 ELISA Kit (HM13272, Bioswamp®) were applied in experiments.

### 2.6. Statistical Analysis

Statistical tests were performed in *R*, unless otherwise mentioned. Spearman's rank correlation coefficient was determined for the fold-change in gene expression and methylation (*β* values). Fisher's exact test was used for comparisons with a small sample size. All *p*values were adjusted using the Benjamini-Hochberg multiple hypothesis testing correction, and an adjusted *p*value <0.05 was considered to be significant. In verification tests, a *t*-test was used to compare the two groups. Statistical significance was set at *p*value <0.05.

## 3. Results

### 3.1. Characterization of Differentially Methylated Probes (DMPs) in Psoriasis as Compared with Healthy Controls

**Figure 1:**
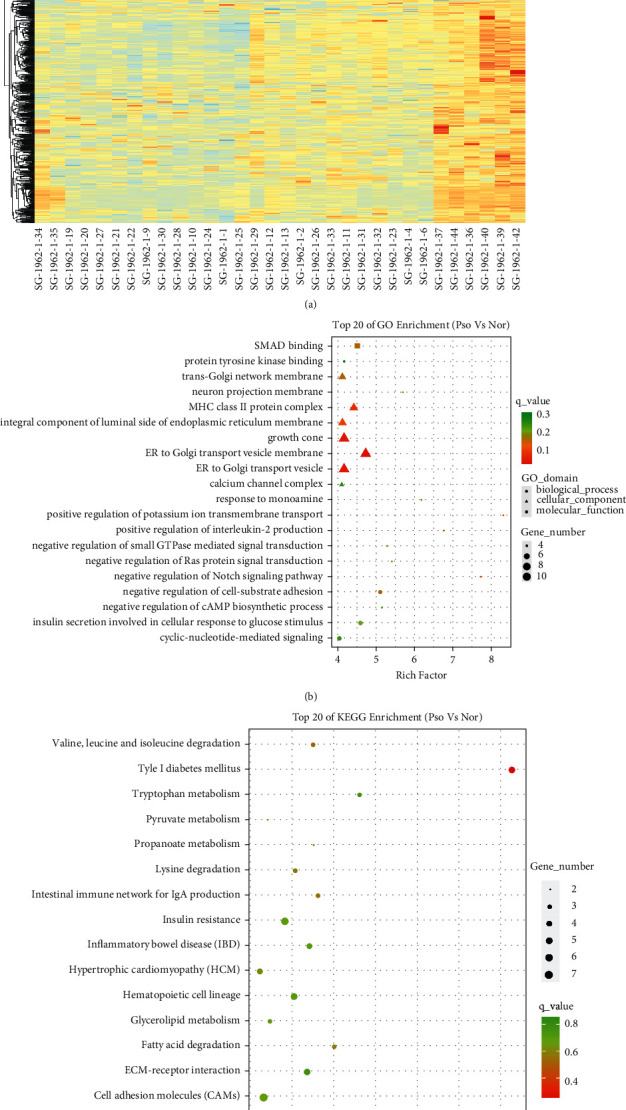
Characterization of differential methylation levels in psoriasis vs. normal. (a) Heat map of hierarchical cluster analysis: the abscissa represents different samples, the ordinate represents DMSs, normal samples are marked in red, and psoriasis samples are marked in green (according to the top 500 DMSs in the absolute value of ^Δ^*β*). (b) Gene ontology (GO) enrichment analysis: each item only displays the top 20 terms that meet the conditions (the top 20 items do not contain a term with only 1 differential gene enrichment). (c) Gene ontology (GO) enrichment analysis: each item only displays the top 20 terms that meet the conditions (the top 20 items do not contain a term with only 1 differential gene enrichment). (d) Kyoto Encyclopedia of Genes and Genomes (KEGG) pathway: each item only displays the top 20 terms that meet the conditions (the top 20 items do not contain a term with only 1 differential gene enrichment).

We screened for DMPs between patients with psoriasis and healthy controls. A total of 875 differential CpG sites were found, of which 48% (*N* = 423) showed hypomethylation and 52% (*N* = 452) showed hypermethylation (Figure S2(a)). In addition, hierarchical cluster analyses determined different classifications between healthy controls and subjects with psoriasis (Figure 1(a)).

The genome can be divided into three regions: promoter, gene body, and 3′ untranslated region (UTR). The promoter region can be further divided into transcription start site (TSS) 200, TSS1500, 5′UTR, and exon regions. A total of 545 differentially methylated CpG sites were found. DMPs were mainly enriched in the gene body region (58.2%). In the promoter region (41.8%), TSS1500s (17.1%) were the most significant, followed by 5′UTR (10.6%) and TSS200 (7.7%), with the least enrichment in the 3′UTR (3.9%) (Figure S2(b)). In addition, DMPs showed a tendency to be hypomethylated in other regions of the genome except 5′UTR and gene body regions (Figure S2(c)).

CGIs can be further subdivided into the N shelf (2–4 kbp upstream of CGIs), N shore (0–2 kbp upstream of CGIs), the CGIs, the S shore (0–2 kbp downstream of CGIs), and the S shelf (2–4 kbp downstream of CGIs). A total of 339 differentially methylated CpG sites were identified. DMPs were mainly enriched in the CGIs (36.9%), followed by the N shore (25.4%) and S shore (10%) regions. The S shelf and S shore regions had the least enrichment in the DMPs (27.7%) (Figure S2(d)). Additionally, DMPs were mainly hypomethylated in the exons regions, but hypermethylation was more prevalent in the S shelf region (Figure S2(c)).

GO enrichment analysis showed that the DMG-related genes in patients with psoriasis particularly involved negative regulation of cell-substrate adhesion, positive regulation of interleukin-2 production, insulin secretion involved in cellular response to glucose stimulus, and negative regulation of Notch signaling pathway. The cellular component was mainly enriched on ER to Golgi transport vesicle membrane, ER to Golgi transport vesicle, integral component of lumenal side of endoplasmic reticulum membrane, MHC class II protein complex, and growth cone. Their molecular function primarily involved SMAD binding and protein tyrosine kinase binding (Figure 1(c)).

KEGG pathway enrichment analysis showed that differentially methylated genes (DMGs) in psoriasis were mainly enriched in the type I diabetes mellitus, autoimmune thyroid disease, bacterial invasion of epithelial cells, ECM-receptor interaction, tryptophan metabolism, insulin resistance, hematopoietic cell lineage, inflammatory bowel disease (IBD), cell adhesion molecules (CAMs), and other pathways (Figure 1(d)).

### 3.2. Characterization of DMPs in Psoriatic BHS and BSS as Compared with Healthy Controls

**Figure 2:**
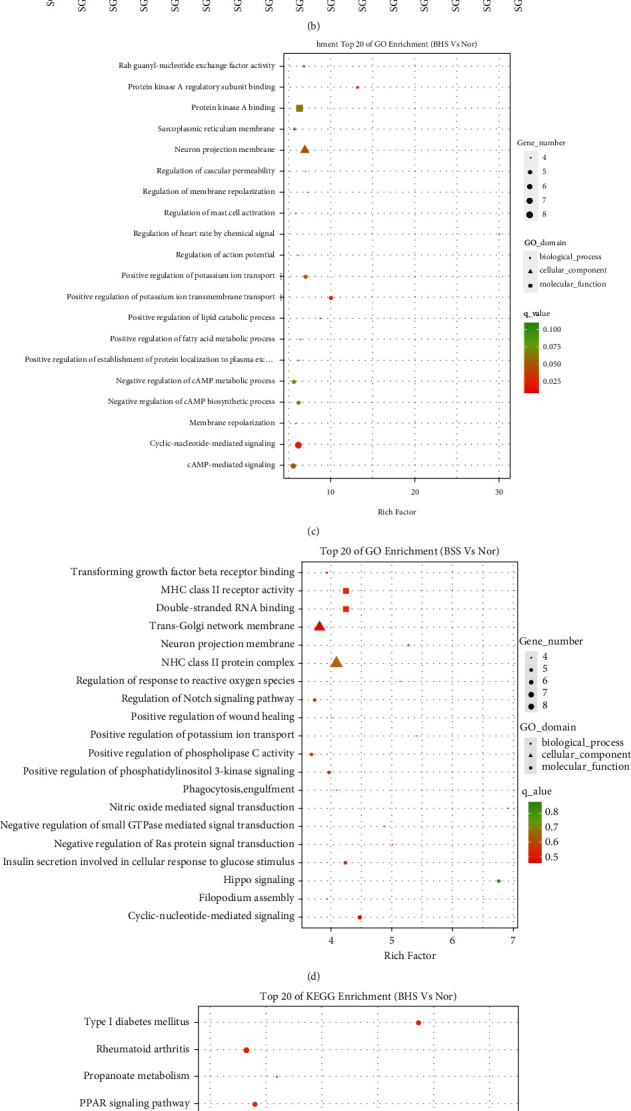
Characterization of differential methylation levels in psoriatic BHS and BSS vs. normal. (a, b) Heat maps representing the hierarchical cluster analysis of BHS and BSS in psoriasis (according to the top 500 DMSs in the absolute value of ^Δ^*β*). (c–f) Gene Ontology (GO) and Kyoto Encyclopedia of Genes and Genomes (KEGG) pathway enrichment analysis: each item only displays the top 20 terms that meet the conditions (the top 20 items do not contain a term with only differential gene enrichment; (c, e) BHS of psoriasis; (d, f) BSS of psoriasis).

A total of 1031 differential CpG sites were found in the BHS, and 750 differential CpG sites were found in the BSS cases. Compared with healthy controls, BHS patients showed hypomethylation, whereas BSS patients were dominated by hypomethylation (Figures S3(a) and S3(b)). Hierarchical cluster analyses could also distinguish the two types of psoriatic syndromes from healthy controls (Figures 2(a) and 2(b)).

Six-hundred and seventy-four differential CpG sites were found to be distributed in the genome of patients with psoriasis BHS, while 705 differential CpG sites were found in the genome of patients with BSS. The DMPs of the two TCM syndromes were mainly enriched in the gene body regions. In the promoter region, DMPs were mainly enriched in the TSS1500 and 5′UTRs regions in BHS and BSS patients. In terms of methylation expression trends in different regions of the genome, both BHS and BSS patients were hypomethylated (Figures S3(c) and S3(d)).

In different regions of CpG islands, specifically in both BHS and BSS, DMPs were mainly enriched in the CGIs, followed by the shore regions, and were least enriched in the shelf regions. In terms of methylation expression trends, except that the N shelf region of BSS patients is hypermethylated, the methylation trends of other regions are the same in BHS and BSS patients, showing hypomethylation (Figures S3(e) and S3(f)).

GO enrichment analysis manifested that, compared with healthy controls, the DMGs of BHS were mainly enriched in the biological processes of positive regulation of potassium ion transport, positive regulation of potassium ion transmembrane transport, cyclic-nucleotide-mediated signaling, and cAMP-mediated signaling. In contrast, the DMGs of BSS were mainly enriched in hippo signaling, response to organic cyclic compound, positive regulation of potassium ion transmembrane transport, antigen processing and presentation of peptide or polysaccharide antigen via MHC class II, and nitric oxide mediated signal transduction (Figures 2(c) and 2(d)).

KEGG pathway enrichment analysis revealed that DMGs of psoriatic BHS were mainly enriched in rheumatoid arthritis, PPAR signaling vpathway, insulin resistance, inflammatory bowel disease (IBD), AMPK signaling pathway, Epstein-Barr virus infection, Herpes simplex infection, and Adherens junction. DMGs of psoriatic BSS were mainly enriched in valine, leucine, and isoleucine degradation, pyruvate metabolism, and tryptophan metabolism, Notch signaling pathway, type I diabetes mellitus, and propanoate metabolism (Figures 2(e) and 2(f)).

### 3.3. Characterization of DMPs in Psoriatic BHS Compared with BSS

**Figure 3:**
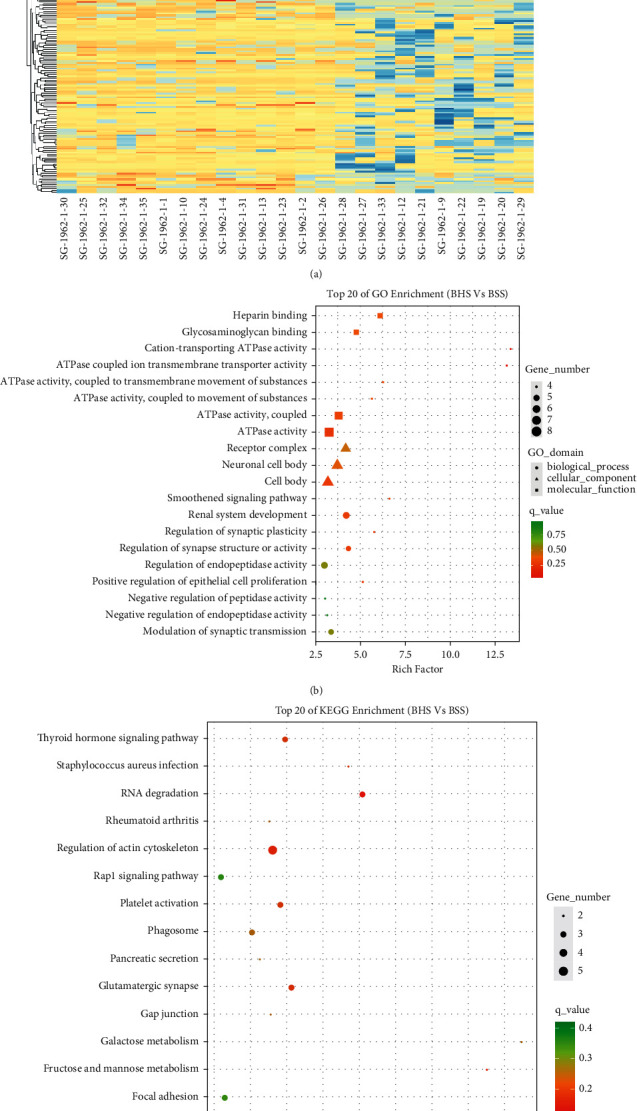
Characterization of differential methylation levels in psoriatic BHS *vs*. psoriatic BSS. (a) Heat map of hierarchical cluster analysis (according to the top 500 DMSs in the absolute value of ^Δ^*β*). (b, c) Gene Ontology (GO) and Kyoto Encyclopedia of Genes and Genomes (KEGG) pathway enrichment analysis: each item only displays the top 20 terms that meet the conditions (the top 20 items do not contain a term with only 1 differential gene enrichment).

Finally, we compared and analyzed the differential sites of psoriatic BHS as well as BSS and found 247 differential CpG sites, of which 53% (*n* = 130) were hypomethylated, together with 47% (*n* = 117) hypermethylated. Hierarchical cluster analysis indicated different classifications of the BHS and BSS samples (Figures 3(a) and S4(a)).

In terms of distribution throughout the genome, 68 CpG sites were identified. DMPs were mainly enriched in the gene body region (33.33%) followed by the 5′UTRs (9.7%) regions. In exonic regions, only 1 differential CpG site was enriched. DMPs were mainly hypermethylated in the TSS1500 and TSS200 regions and hypomethylated in the 3′UTRs regions. In addition, the DMPs in the 3′UTRs, 5′UTRs, and exonic regions were all hypomethylated (Figure S4(b)).

In the analyses of the distribution in CGI regions, we identified 60 differentially methylated CpG sites. DMPs were mainly enriched in the S shelf (26.7%) area, followed by the S shore (23.3%) area, and were less distributed in the island areas 20%. In contrast, the distribution of DMPs in the N shelf (18.3%) and N shore (1.7%) area was the lowest. In addition, in terms of methylation levels, the S shore region was hypermethylated. In other regions, the levels of methylation were similar (Figure S4(c)).

GO enrichment analyses revealed that the DMGs in psoriasis BHS and BSS patients were mainly enriched in the biological processes of regulation of synapse structure or activity, regulation of peptidase activity, regulation of endopeptidase activity, and modulation of synaptic transmission. In terms of cellular components, DMGs were mainly enriched in neuronal cell body, receptor complex, and cell body (Figure 3(b)). Molecular functions were mainly enriched in heparin binding, glycosaminoglycan binding, and ATPase activity. KEGG pathway enrichment analyses manifested that the DMGs in psoriatic BHS and BSS were mainly enriched in platelet activation, complement and coagulation cascades, regulation of actin cytoskeleton, *Staphylococcus aureus* infection, glutamatergic synapse, thyroid hormone signaling pathway, bacterial invasion of epithelial cells, and ECM-receptor interaction (Figure 3(c)).

### 3.4. Correlation Analysis between DNA Methylation Level and PASI Score of Psoriasis

**Figure 4:**
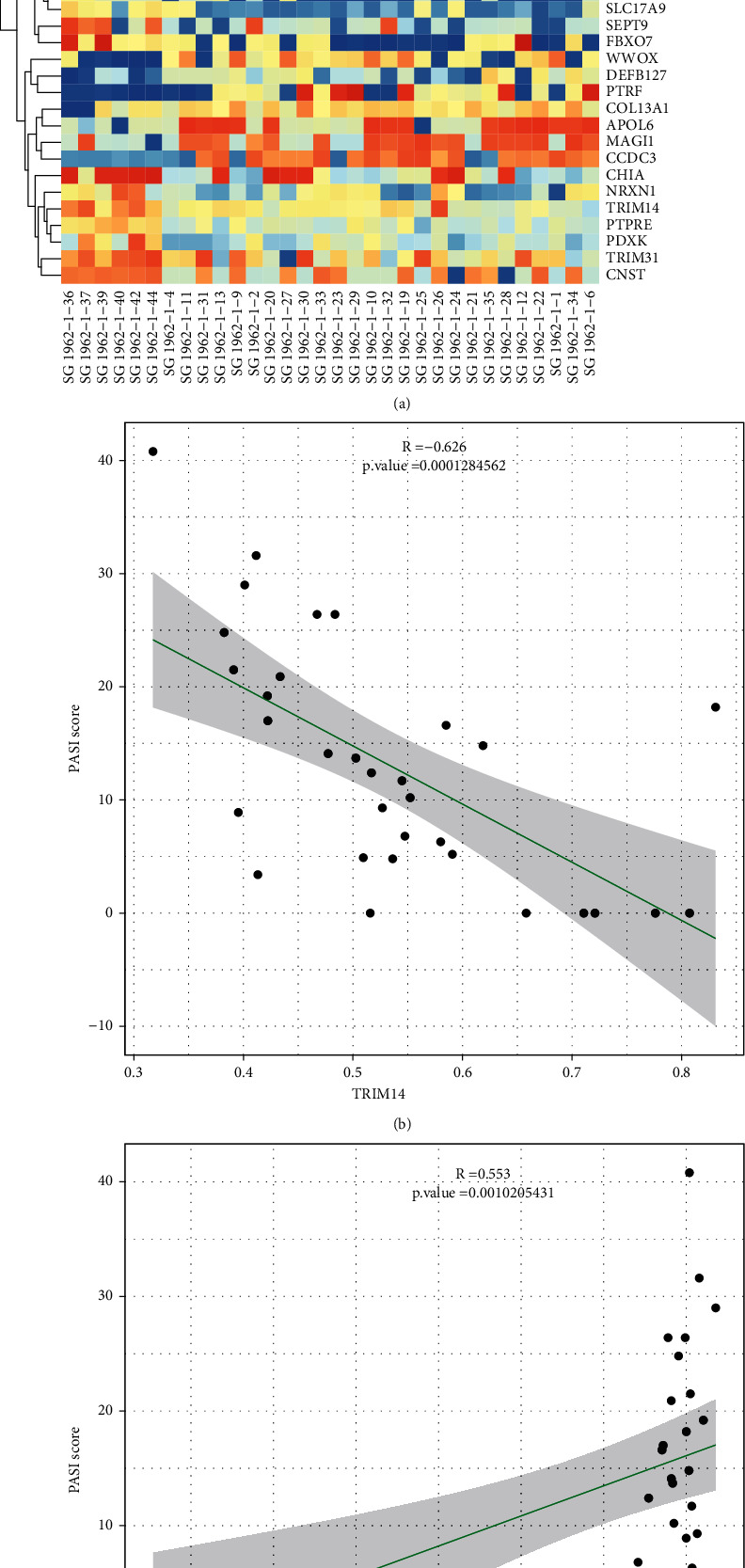
Spearman's correlation analysis between differential methylation probe (DMG) methylation level and psoriasis area and severity index (PASI) score. (a) Blue and red represent different levels of methylation, from low to high. The green color represents the PASI score: the darker the color, the higher the score (according to all DMGs with *p*value ≤ 0.01 and |beta difference| and |R value| ≥ 2. (b) These genes represent the maximum and minimum values of *R*, respectively.

Finally, we screened out the DMGs related to the PASI score of psoriasis using Spearman's correlation analysis. A total of 483 DMGs were identified in which the level of methylation correlated with the PASI score. To improve the accuracy and rigor of the data, we set the *p* value ≤ 0.01 and the |beta difference| and |*R* value | ≥ 2 for screening and obtained 42 DMGs, of which 21 DMGs were negatively correlated with disease severity and 16 DMGs were positively correlated with disease severity (Figure 4(a)).

### 3.5. Experimental Verification

**Figure 5:**
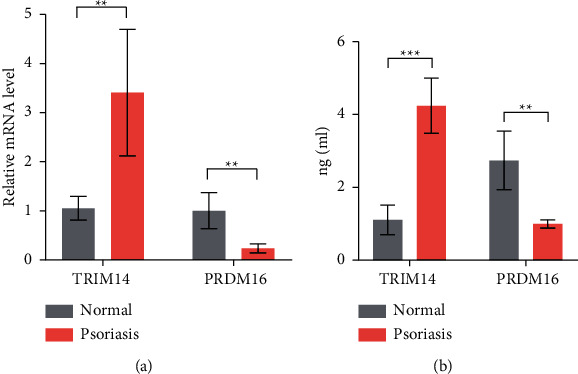
Expression of TRIM14 and PRDM16 in peripheral blood mononuclear cells (PBMC) of psoriasis vs. normal. (a) The expression of TRIM14 and PRDM16 mRNA was determined by RT-PCR. (b) TRIM14 and PRDM16 protein expression was analyzed by ELISA. ^*∗*^*p* < 0.05, ^*∗∗*^*p* < 0.01, and ^*∗∗∗*^*p* < 0.001, compared with the normal group (*n* = 5).

In the correlation analysis between psoriasis methylation level and PASI score, the highest negative correlation was *TRIM14*, and the highest positive correlation was *PRDM16* (Figure 4(b)). DNA methylation sequencing results suggest that *TRIM14* is hypomethylated in psoriasis, whereas *PRDM16* is hypermethylated. For further verification, we found that the expression of *TRIM14* mRNA increased and *PRDM16* mRNA decreased in PBMC of psoriasis patients by RT-PCR (Figure 5(a)) and the protein expression detected by ELISA showed the same trend (Figure 5(b)). Therefore, we speculate that DNA methylation modifications are involved in the expression of *TRIM14* and *PRDM16* in psoriasis.

## 4. Discussion

In recent years, with the advancement of psoriasis research, methylation modification has gradually received attention. Methylation modification mainly involves DNA and RNA. DNA methylation modification mainly works at the transcription level, while RNA methylation mainly regulates gene expression at the posttranscriptional level. DNA methylation has been confirmed to play an important role in the pathogenesis of many autoimmune skin diseases associated with psoriasis, such as systemic lupus erythematosus, dermatomyositis, and scleroderma [12]. At present, most studies on DNA methylation in psoriasis have focused on skin tissues [2, 13]. However, as a systemic autoimmune disease, the skin may be the ultimate target of a series of immune mechanism interactions. Therefore, the treatment of moderate and severe psoriasis is still based on systemic treatment [14]. PBMCs include lymphocytes (T and B), monocytes, phagocytes, dendritic cells, and a small number of other cell types. They are the main components of immune cells and play an important role in the immune response of the body. Some studies have found that the expression of many differentially expressed genes in psoriatic skin samples is related to inflammation, which is a systemic rather than an organ-specific phenomenon. In contrast, the expression of differential genes in PBMCs is more specific than that in skin tissue [15].

Currently, the Methylamp Global DNA Methylation Quantification Kit is used to assess the overall methylation trend in PBMCs of patients with psoriasis. A previous study found that, compared with healthy controls, the overall methylation trend in psoriasis is increased significantly [16]. However, global DNA methylation can only understand the changes in the overall DNA methylation level but cannot highlight changes at specific sites. In contrast, whole-genome analyses of skin tissues revealed that psoriatic samples were hypomethylated [17]. Therefore, the DNA methylation level of psoriasis in PBMCs requires further verification and in-depth analyses.

In this study, we used the Illumina 850k methylation chip, which can detect the methylation status of approximately 853,307 CpG sites in the human genome and contains more than 90% of the original 450k chip. Through comparison of DNA methylation in PBMCs between psoriatic patients and healthy controls, we found that the DMSs in PBMCs of patients with psoriasis mainly involved hypermethylation (52%) and further divided psoriasis into two TCM syndromes: the BHS and BSS. Compared with healthy controls, the DMSs show hypermethylation in BHS and hypomethylation in BSS. The DMPs of psoriatic patients were mainly distributed in the gene body (58.2%) and TSS1500 (17.1%) regions of the genome. In psoriasis, BHS and BSS showed the same distribution trend. CGIs are defined as regions with more than 200 base pairs and more than 50% GC content. They are often located near the promoters of genes and participate in regulating gene expression. We divided CpGs into CGI, shelf, and shore regions. DMPs were significantly enriched in CGIs and mainly involved hypomethylation in both groups.

Biofunctional informatics revealed differences in the biological processes related to DMGs between different syndromes of psoriasis and healthy controls. We also performed KEGG pathway enrichment analysis on DMGs of psoriasis patients and found that they were mainly enriched in type I diabetes mellitus, autoimmune thyroid disease, bacterial invasion of epithelial cells, ECM-receptor interaction, tryptophan metabolism, insulin resistance, hematopoietic cell lineage, inflammatory bowel disease (IBD), and cell adhesion molecules (CAMs), among others. The DMGs of BHS psoriasis were mainly enriched in rheumatoid arthritis, PPAR signaling pathway, insulin resistance, inflammatory bowel disease (IBD), AMPK signaling pathway, and adherent junction. In BSS psoriasis, there was mainly enrichment in valine, leucine, and isoleucine degradation, pyruvate metabolism, and tryptophan metabolism, Notch signaling pathway, type I diabetes mellitus, and propanoate metabolism.

TCM holds that psoriatic BHS is the progressive stage of the disease, while psoriasis BSS is the quiescent stage of the disease. Interestingly, we found that the DMGs of psoriasis BHS were enriched in Epstein-Barr virus infection and Herpes simplex infection. Respiratory virus infections are important factors that induce psoriasis [18]. In addition, there is evidence that Herpes simplex virus infection is a potential risk factor for psoriasis [19]. The BSS of psoriasis indicates that the disease has entered a chronic stage; TCM holds that the disease changes from acute to chronic because, in the process of struggle between healthy qi and pathogenic factors, the healthy qi is damaged by pathogenic factors. The immune system then becomes unable to drive out the pathogenic factors while weakened pathogenic factors cannot further aggravate the disease, resulting in a nonintense but persistent pathological state. In clinical treatment, while improving blood circulation and dispersing stasis, the application of Chinese herbs with a tonic effect is often emphasized. The DMGs of BSS are enriched in the valine, leucine, and isoleucine degradation, pyruvate metabolism, and tryptophan metabolism, which are the building blocks of proteins needed for human nutrition reminiscent of the TCM concept and treatment principle of strengthening the body's resistance to chronic diseases.

BHS and BSS are unique pathological concepts of TCM. We analyzed the differential DNA methylation sites between the two syndrome types and identified 247 different methylation sites mainly involving hypomethylation. However, the distribution of these sites in the genome was different among groups. GO function analyses of DMGs pointed to wound healing, blood coagulation hemostasis, and coagulation. KEGG pathway analyses highlighted platelet activation, complement, and coagulation cascade. TCM believes that BHS refers to heat entering the blood, which often forces the blood flow to be unconstrained. BSS is caused by irregular blood circulation, blocked meridians, and blood stasis. Ultrasound studies have shown that, compared with BSS in psoriasis, the arterial and venous blood vessels under the skin lesions of BHS patients and healthy skin are enlarged and dilated and blood flow is accelerated [20]. In contrast, a large number of studies have confirmed that BSS is generally closely related to circulatory or microcirculation disorders, which leads to abnormal blood rheology [21–23].

In the correlation analysis between DNA methylation level and psoriasis severity, we found that *TRIM14* gene was significantly and negatively correlated, while *PRDM16* gene was significantly and positively correlated. The tripartite motif (TRIM) family of proteins consists of a RING domain (R), two B-box domains (B1 and B2), and a coiled-coil (CC) region [24]. *TRIM14* is a member of the *TRIM* family and is located at chromosome 9q22. The NF-*κ*B signaling pathway is an important link in the immune and inflammatory response of psoriasis [25, 26]. Studies have shown that overexpression of *TRIM14* promotes the phosphorylation and degradation of *IκBα* induced by *TNF-α* and activates the NF-*κ*B signaling pathway [27]. The STAT3 signaling pathway plays an important role in the differentiation of psoriatic TH17 cells [28], and TRIM14 also positively regulated the protein levels of phosphorylated *STAT3* (p-STAT3), as well as the mRNA and protein expression of matrix metalloproteinase 2, *MMP9*, and vascular endothelial growth factor, which are transcriptional targets of the STAT3 signaling pathway [29]. Psoriasis is closely related to metabolic syndrome [30], and a large amount of clinical evidence has confirmed that the incidence of diabetes and obesity in patients with psoriasis is much higher than that in healthy people [31, 32]. Clinical and basic studies have shown that the expression of *PRDM16* is associated with obesity and diabetes and that PRDM16 signaling participates in the treatment of the two diseases [33, 34]. In addition, *PRDM16* overexpressing mice presented increased energy expenditure, limited weight gain, improved glucose tolerance, and responded to a high-fat diet. Therefore, *PRDM16* may play an important role in the pathogenesis of psoriasis. Our study found that methylation levels of genes *TRIM14* and *PRDM16* may be important markers of psoriasis severity, but the specific mechanism of action of these associated genes requires further research.

## 5. Conclusions

In summary, in the present study, we found that DNA methylation in PBMCs of psoriasis showed a trend of hypermethylation. DMGs involve a variety of inflammatory signal pathways and are closely related to a variety of immune and metabolic diseases. Our work also revealed that there are different sites of DNA methylation between BHS and BSS of psoriasis and the genes involved in related sites have different biological functions, indicating the different mechanism between TCM syndrome types of psoriasis. In addition, DNA methylation levels of *TRIM14* and *PRDM16* may serve as potential biomarkers for assessing the severity of psoriasis.

## Data Availability

The data generated from this article can be found in the Gene Expression Omnibus database (https://www.ncbi.nlm.nih.gov/geo/), using accession number GSE183608.

## Supplementary Materials

Supplementary Materials
*Table S1*. It included subject information. *Table S2.* Diagnostic criteria of blood heat syndrome (BHS) and blood stasis syndrome (BSS). *Table S3*. TREND statement checklist. *Figure S1*. Sample quality control chart note. The horizontal axis is log2 (methylated median value) and the vertical axis is nonmethylated median value. The overall methylation degree of each sample is represented by a dot, and its distribution in the upper right corner of the dotted line indicated that the quality control standard had been met. All samples in this study met the standard of quality control. *Figure S2.* Characterization of DMPs in psoriasis *vs*. normal (*N* = 875). (a) Orange and green represent the proportion of hypermethylated and hypomethylated DMPs, respectively. (b) Distribution of DMPs in different regions of the genome. (c) Distribution of DMPs in the genome and CpG island regions. (d) Distribution of methylation levels in different regions of genome and CpG islands. *Figure S3.* Characterization of DMPs in psoriatic BHS (*N* = 1031) and BSS (*N* = 1094) vs. normal. (a, b) Orange and green represent the proportion of hypermethylated and hypomethylated DMPs of BHS vs. normal (left) and BSS vs. normal (right), respectively. (c) DMPs in different regions of the genome in psoriasis with BHS. (d) Distribution of DMPs in different regions of the genome in psoriasis patients with BSS. (e) Distribution of DMPs in different regions of CpG islands in psoriasis patients with BHS. (f) Distribution of DMPs in different regions of CpG islands in psoriasis patients with BSS. (g, h) Distribution of methylation levels in different regions of genome and CpG islands. *Figure S4.* Characterization of DMPs in psoriatic BHS vs. psoriatic BSS (*N* = 247). (a) Orange and green represent the proportion of hypermethylated and hypomethylated DMPs, respectively. (b) Distribution of DMPs in different regions of the genome. (c) Distribution of DMPs in different regions of CpG islands. (d) Distribution of methylation levels in different regions of genome and CpG islands.
