# Supplementary material for: DNA Methylation Expression Profile of Blood Heat Syndrome and Blood Stasis Syndrome in TCM Psoriasis
Source: Evid Based Complement Alternat Med. 2022 Sep 19;2022:9343285. doi: 10.1155/2022/9343285 (PMC9526661; doi:10.1155/2022/9343285)
Supplement: Supplementary Materials — Table S1. It included subject information. Table S2. Diagnostic criteria of blood heat syndrome (BHS) and blood stasis syndrome (BSS). Table S3. TREND statement checklist. Figure S1. Sample quality control chart note. The horizontal axis is log2 (methylated median value) and the vertical axis is nonmethylated median value. The overall methylation degree of each sample is represented by a dot, and its distribution in the upper right corner of the dotted line indicated that the quality control standard had been met. All samples in this study met the standard of quality control. Figure S2. Characterization of DMPs in psoriasis vs. normal (N = 875). (a) Orange and green represent the proportion of hypermethylated and hypomethylated DMPs, respectively. (b) Distribution of DMPs in different regions of the genome. (c) Distribution of DMPs in the genome and CpG island regions. (d) Distribution of methylation levels in different regions of genome and CpG islands. Figure S3. Characterization of DMPs in psoriatic BHS (N = 1031) and BSS (N = 1094) vs. normal. (a, b) Orange and green represent the proportion of hypermethylated and hypomethylated DMPs of BHS vs. normal (left) and BSS vs. normal (right), respectively. (c) DMPs in different regions of the genome in psoriasis with BHS. (d) Distribution of DMPs in different regions of the genome in psoriasis patients with BSS. (e) Distribution of DMPs in different regions of CpG islands in psoriasis patients with BHS. (f) Distribution of DMPs in different regions of CpG islands in psoriasis patients with BSS. (g, h) Distribution of methylation levels in different regions of genome and CpG islands. Figure S4. Characterization of DMPs in psoriatic BHS vs. psoriatic BSS (N = 247). (a) Orange and green represent the proportion of hypermethylated and hypomethylated DMPs, respectively. (b) Distribution of DMPs in different regions of the genome. (c) Distribution of DMPs in different regions of CpG islands. (d) Distribution of methylation l [file 9343285.f1.zip › 9343285.f1/Table S1-3 (1).docx]

**Table S1. Included subject information**

| **No.** | **Group** | **Name** | **Sex** | **Age** | **PASI score** |
| --- | --- | --- | --- | --- | --- |
| SG 1962-1-1 | BHS | GFL | M | 35 | 29 |
| SG 1962-1-2 | BHS | YCP | F | 65 | 6.8 |
| SG 1962-1-4 | BHS | ZYB | M | 43 | 3.4 |
| SG 1962-1-10 | BHS | LY | F | 24 | 14.1 |
| SG 1962-1-13 | BHS | JZQ | M | 19 | 5.2 |
| SG 1962-1-23 | BHS | XLJ | M | 33 | 12.4 |
| SG 1962-1-24 | BHS | FTY | F | 20 | 19.2 |
| SG 1962-1-25 | BHS | XHM | M | 58 | 17 |
| SG 1962-1-26 | BHS | YJ | F | 31 | 18.2 |
| SG 1962-1-30 | BHS | CCT | M | 31 | 10.2 |
| SG 1962-1-31 | BHS | XH | F | 41 | 4.9 |
| SG 1962-1-32 | BHS | LH | F | 45 | 14.8 |
| SG 1962-1-34 | BHS | SML | F | 38 | 31.6 |
| SG 1962-1-35 | BHS | TX | M | 31 | 21.5 |
| SG 1962-1-6 | BSS | CSM | M | 57 | 40.8 |
| SG 1962-1-9 | BSS | LWB | M | 45 | 6.3 |
| SG 1962-1-11 | BSS | XJY | F | 45 | 4.8 |
| SG 1962-1-12 | BSS | ZJX | M | 18 | 26.4 |
| SG 1962-1-19 | BSS | HQM | M | 56 | 16.6 |
| SG 1962-1-20 | BSS | SF | F | 36 | 8.9 |
| SG 1962-1-21 | BSS | YWM | F | 65 | 20.9 |
| SG 1962-1-22 | BSS | ZJH | F | 17 | 26.4 |
| SG 1962-1-27 | BSS | ZJX | M | 31 | 9.3 |
| SG 1962-1-28 | BSS | GHJ | F | 44 | 24.8 |
| SG 1962-1-29 | BSS | ZSD | M | 62 | 13.7 |
| SG 1962-1-33 | BSS | LHQ | F | 37 | 11.7 |
| SG 1962-1-44 | Normal | TJ | M | 41 | NA |
| SG 1962-1-40 | Normal | YRG | F | 35 | NA |
| SG 1962-1-42 | Normal | ZQ | M | 47 | NA |
| SG 1962-1-36 | Normal | ZXY | F | 45 | NA |
| SG 1962-1-37 | Normal | ZL | F | 43 | NA |
| SG 1962-1-39 | Normal | WWC | M | 34 | NA |

BHS: blood heat syndrome; BSS: blood stasis syndrome; F: Female; M: Male; NA: not applicable.

**Table S2. Diagnostic criteria of psoriasis with Blood heat syndrome (BHS) and blood stasis syndrome (BSS)**

|  | **Psoriasis with BHS** | **Psoriasis with BSS** |
| --- | --- | --- |
| **Main symptoms** | ① Skin lesions are bright red.  ② New rashes are increasing in number or rapidly expanding. | ① Skin lesions are dark red.  ② Skin lesions are often hypertrophic, often accompanied by infiltration, showing a trend of chronic recurrence. |
| **Secondary symptoms** | ① Upset and irritable mood.  ② Yellow urine.  ③ Red or crimson tongue.  ④ The pulse string is slippery or counting. | ① Rough skin with scaly, dark complexion, or bruising of lips and nails.  ② Women's menstrual blood is dark, or there are blood clots.  ③ The tongue is dark purple or has petechiae or ecchymosis.  ④ The pulse is astringent or sluggish. |
| **Confirmed** | Confirmed: Presence of all main symptoms and one or more secondary symptoms. | Confirmed: Presence of all main symptoms and one or more secondary symptoms. |

**Table S3. Comparison of p value with included subject information**

| **Groups vs.** | **Sex** | **Age** |
| --- | --- | --- |
| Psoriasis vs. Normal | 0.673 | 0.827 |
| BHS vs. Normal | 0.686 | 0.473 |
| BSS vs. Normal | 0.690 | 0.779 |
| BHS vs. BSS | 0.652 | 0.298 |

Vs.: versus; BHS: blood heat syndrome; BSS: blood stasis syndrome.
