# Supplementary figures and images for: DNA Methylation Expression Profile of Blood Heat Syndrome and Blood Stasis Syndrome in TCM Psoriasis
Source: Evid Based Complement Alternat Med. 2022 Sep 19;2022:9343285. doi: 10.1155/2022/9343285 (PMC9526661; doi:10.1155/2022/9343285)

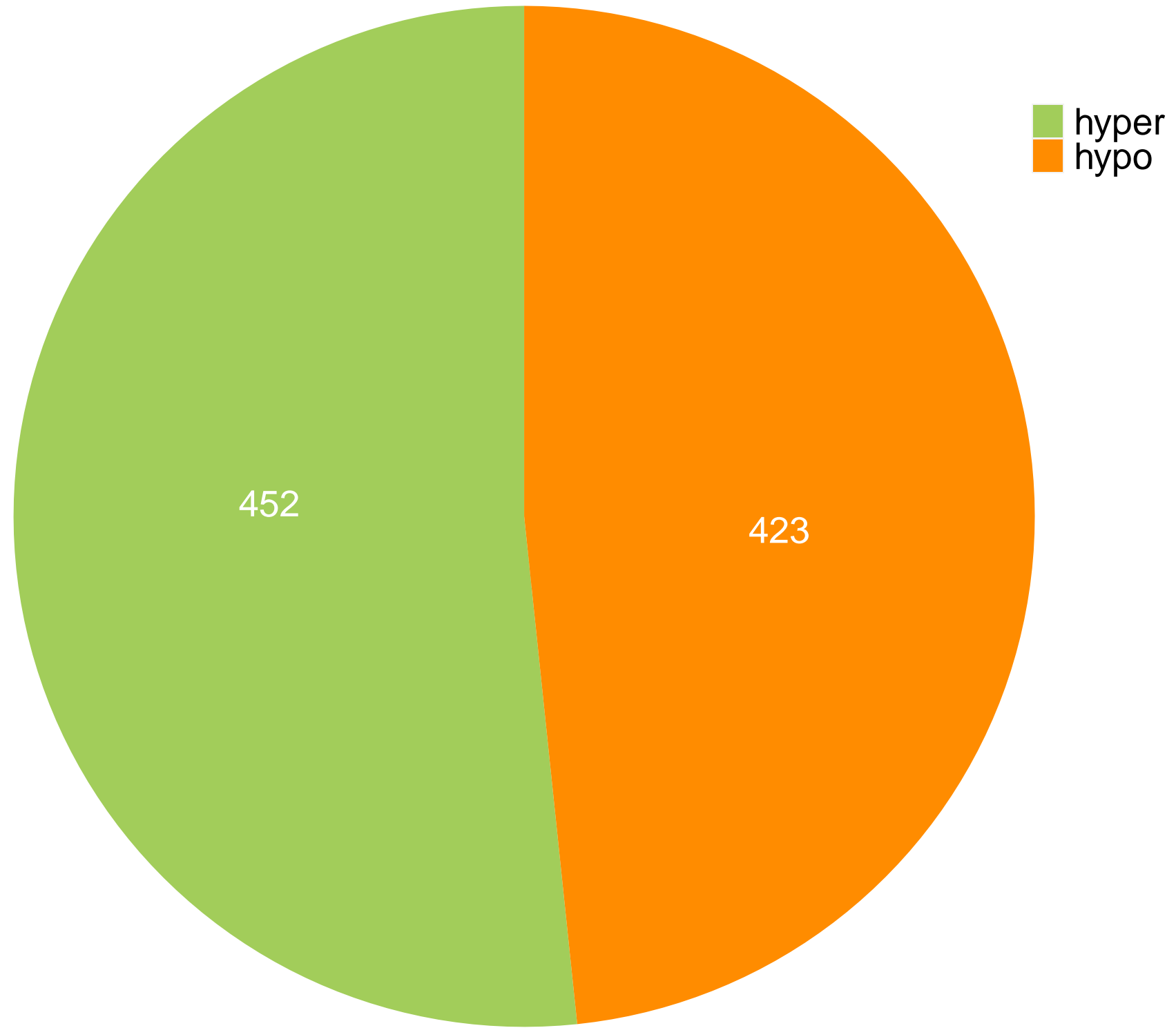

Supplement: Supplementary Materials — Table S1. It included subject information. Table S2. Diagnostic criteria of blood heat syndrome (BHS) and blood stasis syndrome (BSS). Table S3. TREND statement checklist. Figure S1. Sample quality control chart note. The horizontal axis is log2 (methylated median value) and the vertical axis is nonmethylated median value. The overall methylation degree of each sample is represented by a dot, and its distribution in the upper right corner of the dotted line indicated that the quality control standard had been met. All samples in this study met the standard of quality control. Figure S2. Characterization of DMPs in psoriasis vs. normal (N = 875). (a) Orange and green represent the proportion of hypermethylated and hypomethylated DMPs, respectively. (b) Distribution of DMPs in different regions of the genome. (c) Distribution of DMPs in the genome and CpG island regions. (d) Distribution of methylation levels in different regions of genome and CpG islands. Figure S3. Characterization of DMPs in psoriatic BHS (N = 1031) and BSS (N = 1094) vs. normal. (a, b) Orange and green represent the proportion of hypermethylated and hypomethylated DMPs of BHS vs. normal (left) and BSS vs. normal (right), respectively. (c) DMPs in different regions of the genome in psoriasis with BHS. (d) Distribution of DMPs in different regions of the genome in psoriasis patients with BSS. (e) Distribution of DMPs in different regions of CpG islands in psoriasis patients with BHS. (f) Distribution of DMPs in different regions of CpG islands in psoriasis patients with BSS. (g, h) Distribution of methylation levels in different regions of genome and CpG islands. Figure S4. Characterization of DMPs in psoriatic BHS vs. psoriatic BSS (N = 247). (a) Orange and green represent the proportion of hypermethylated and hypomethylated DMPs, respectively. (b) Distribution of DMPs in different regions of the genome. (c) Distribution of DMPs in different regions of CpG islands. (d) Distribution of methylation l [file 9343285.f1.zip › 9343285.f1/Figure S2A.pdf]

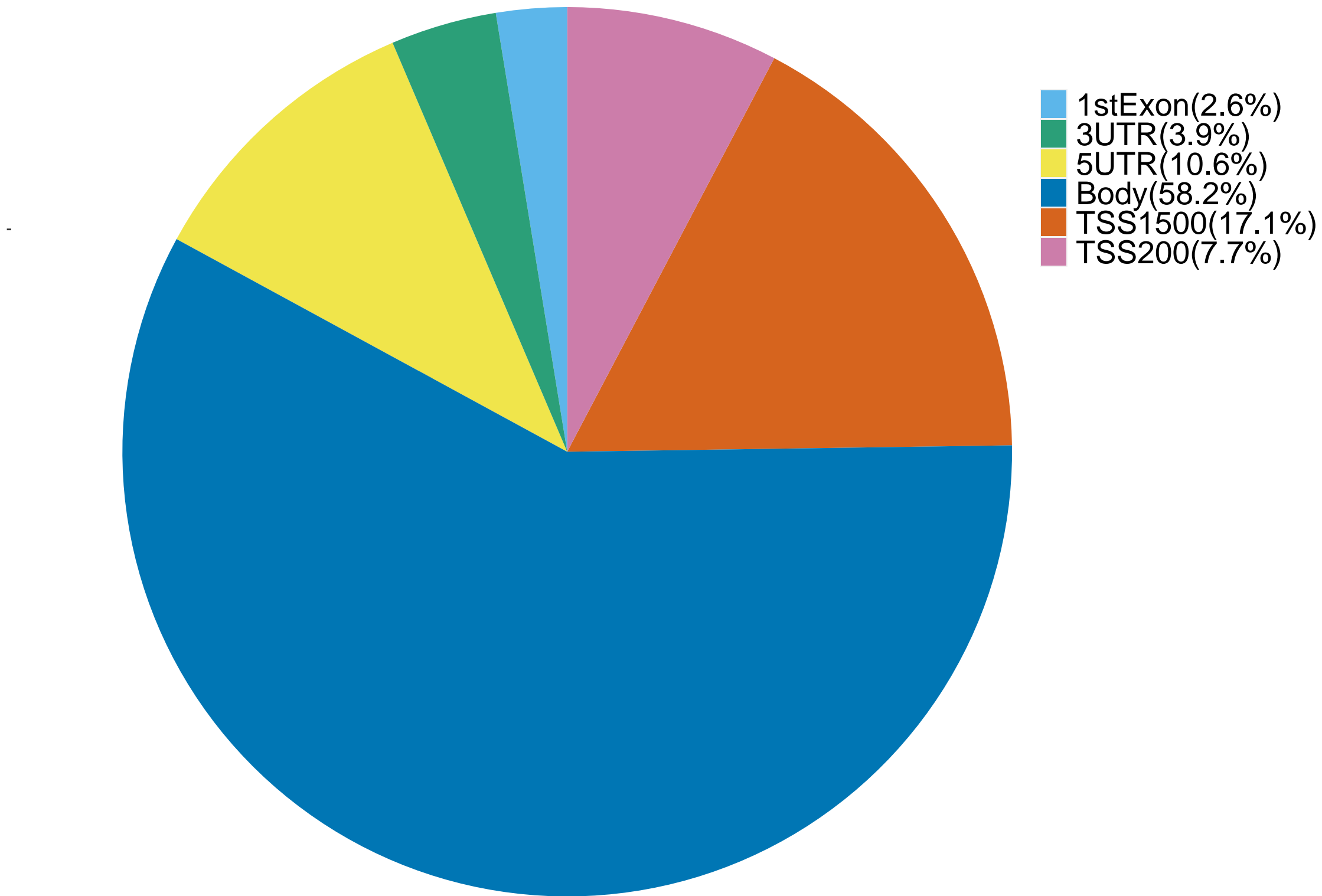

Supplement: Supplementary Materials — Table S1. It included subject information. Table S2. Diagnostic criteria of blood heat syndrome (BHS) and blood stasis syndrome (BSS). Table S3. TREND statement checklist. Figure S1. Sample quality control chart note. The horizontal axis is log2 (methylated median value) and the vertical axis is nonmethylated median value. The overall methylation degree of each sample is represented by a dot, and its distribution in the upper right corner of the dotted line indicated that the quality control standard had been met. All samples in this study met the standard of quality control. Figure S2. Characterization of DMPs in psoriasis vs. normal (N = 875). (a) Orange and green represent the proportion of hypermethylated and hypomethylated DMPs, respectively. (b) Distribution of DMPs in different regions of the genome. (c) Distribution of DMPs in the genome and CpG island regions. (d) Distribution of methylation levels in different regions of genome and CpG islands. Figure S3. Characterization of DMPs in psoriatic BHS (N = 1031) and BSS (N = 1094) vs. normal. (a, b) Orange and green represent the proportion of hypermethylated and hypomethylated DMPs of BHS vs. normal (left) and BSS vs. normal (right), respectively. (c) DMPs in different regions of the genome in psoriasis with BHS. (d) Distribution of DMPs in different regions of the genome in psoriasis patients with BSS. (e) Distribution of DMPs in different regions of CpG islands in psoriasis patients with BHS. (f) Distribution of DMPs in different regions of CpG islands in psoriasis patients with BSS. (g, h) Distribution of methylation levels in different regions of genome and CpG islands. Figure S4. Characterization of DMPs in psoriatic BHS vs. psoriatic BSS (N = 247). (a) Orange and green represent the proportion of hypermethylated and hypomethylated DMPs, respectively. (b) Distribution of DMPs in different regions of the genome. (c) Distribution of DMPs in different regions of CpG islands. (d) Distribution of methylation l [file 9343285.f1.zip › 9343285.f1/Figure S2B.pdf]

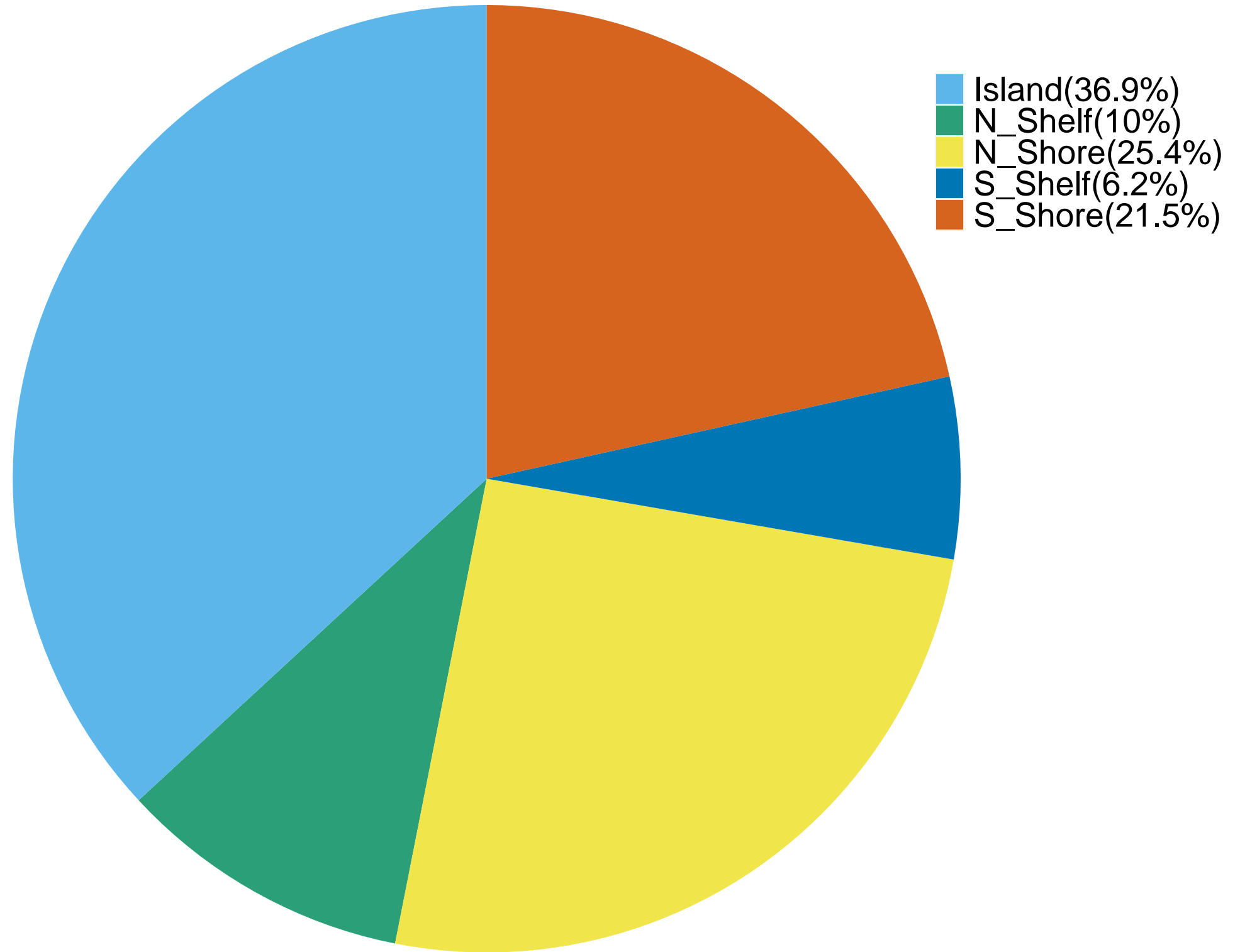

Supplement: Supplementary Materials — Table S1. It included subject information. Table S2. Diagnostic criteria of blood heat syndrome (BHS) and blood stasis syndrome (BSS). Table S3. TREND statement checklist. Figure S1. Sample quality control chart note. The horizontal axis is log2 (methylated median value) and the vertical axis is nonmethylated median value. The overall methylation degree of each sample is represented by a dot, and its distribution in the upper right corner of the dotted line indicated that the quality control standard had been met. All samples in this study met the standard of quality control. Figure S2. Characterization of DMPs in psoriasis vs. normal (N = 875). (a) Orange and green represent the proportion of hypermethylated and hypomethylated DMPs, respectively. (b) Distribution of DMPs in different regions of the genome. (c) Distribution of DMPs in the genome and CpG island regions. (d) Distribution of methylation levels in different regions of genome and CpG islands. Figure S3. Characterization of DMPs in psoriatic BHS (N = 1031) and BSS (N = 1094) vs. normal. (a, b) Orange and green represent the proportion of hypermethylated and hypomethylated DMPs of BHS vs. normal (left) and BSS vs. normal (right), respectively. (c) DMPs in different regions of the genome in psoriasis with BHS. (d) Distribution of DMPs in different regions of the genome in psoriasis patients with BSS. (e) Distribution of DMPs in different regions of CpG islands in psoriasis patients with BHS. (f) Distribution of DMPs in different regions of CpG islands in psoriasis patients with BSS. (g, h) Distribution of methylation levels in different regions of genome and CpG islands. Figure S4. Characterization of DMPs in psoriatic BHS vs. psoriatic BSS (N = 247). (a) Orange and green represent the proportion of hypermethylated and hypomethylated DMPs, respectively. (b) Distribution of DMPs in different regions of the genome. (c) Distribution of DMPs in different regions of CpG islands. (d) Distribution of methylation l [file 9343285.f1.zip › 9343285.f1/Figure S2C.pdf]

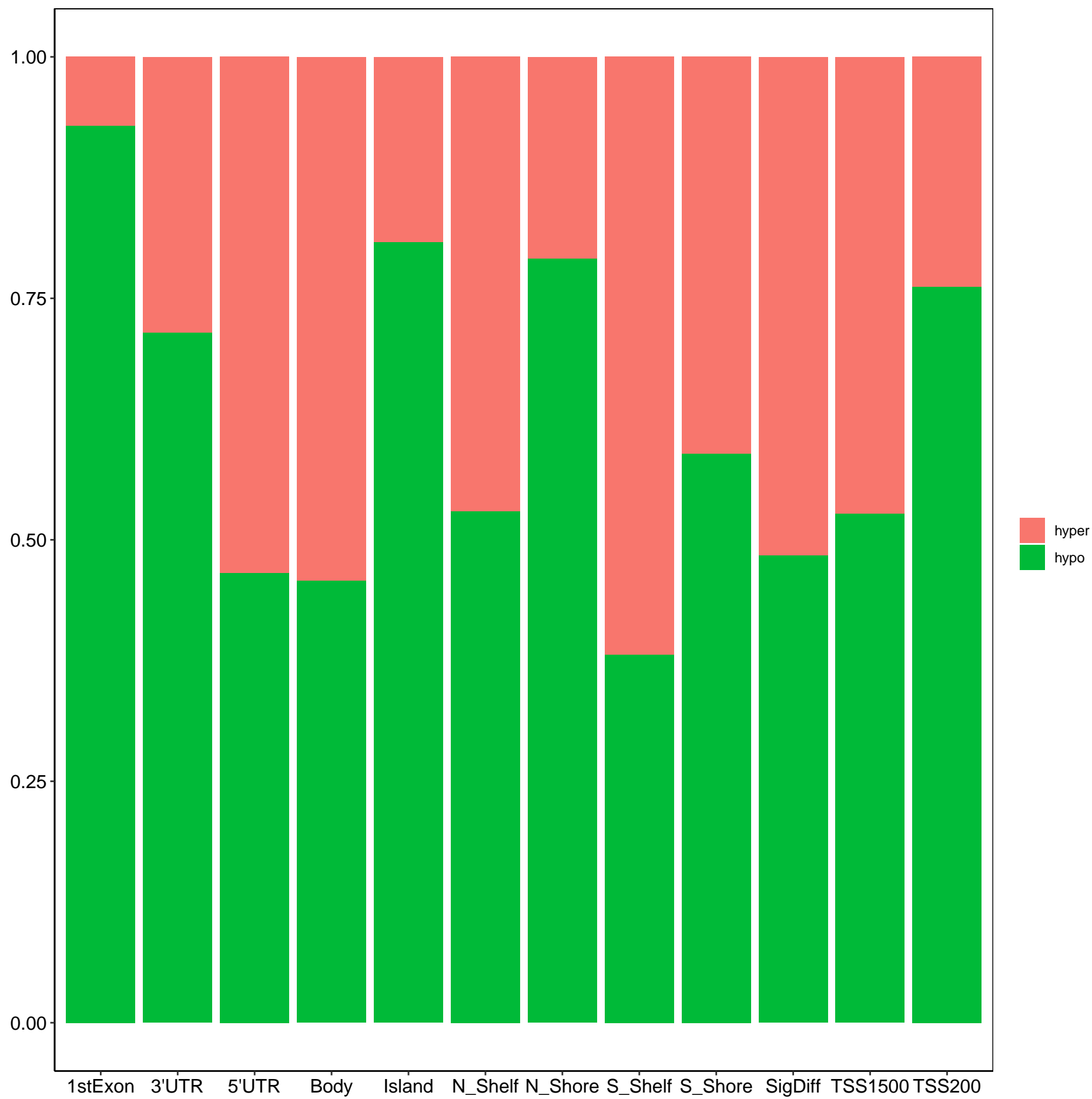

Supplement: Supplementary Materials — Table S1. It included subject information. Table S2. Diagnostic criteria of blood heat syndrome (BHS) and blood stasis syndrome (BSS). Table S3. TREND statement checklist. Figure S1. Sample quality control chart note. The horizontal axis is log2 (methylated median value) and the vertical axis is nonmethylated median value. The overall methylation degree of each sample is represented by a dot, and its distribution in the upper right corner of the dotted line indicated that the quality control standard had been met. All samples in this study met the standard of quality control. Figure S2. Characterization of DMPs in psoriasis vs. normal (N = 875). (a) Orange and green represent the proportion of hypermethylated and hypomethylated DMPs, respectively. (b) Distribution of DMPs in different regions of the genome. (c) Distribution of DMPs in the genome and CpG island regions. (d) Distribution of methylation levels in different regions of genome and CpG islands. Figure S3. Characterization of DMPs in psoriatic BHS (N = 1031) and BSS (N = 1094) vs. normal. (a, b) Orange and green represent the proportion of hypermethylated and hypomethylated DMPs of BHS vs. normal (left) and BSS vs. normal (right), respectively. (c) DMPs in different regions of the genome in psoriasis with BHS. (d) Distribution of DMPs in different regions of the genome in psoriasis patients with BSS. (e) Distribution of DMPs in different regions of CpG islands in psoriasis patients with BHS. (f) Distribution of DMPs in different regions of CpG islands in psoriasis patients with BSS. (g, h) Distribution of methylation levels in different regions of genome and CpG islands. Figure S4. Characterization of DMPs in psoriatic BHS vs. psoriatic BSS (N = 247). (a) Orange and green represent the proportion of hypermethylated and hypomethylated DMPs, respectively. (b) Distribution of DMPs in different regions of the genome. (c) Distribution of DMPs in different regions of CpG islands. (d) Distribution of methylation l [file 9343285.f1.zip › 9343285.f1/Figure S2D.pdf]

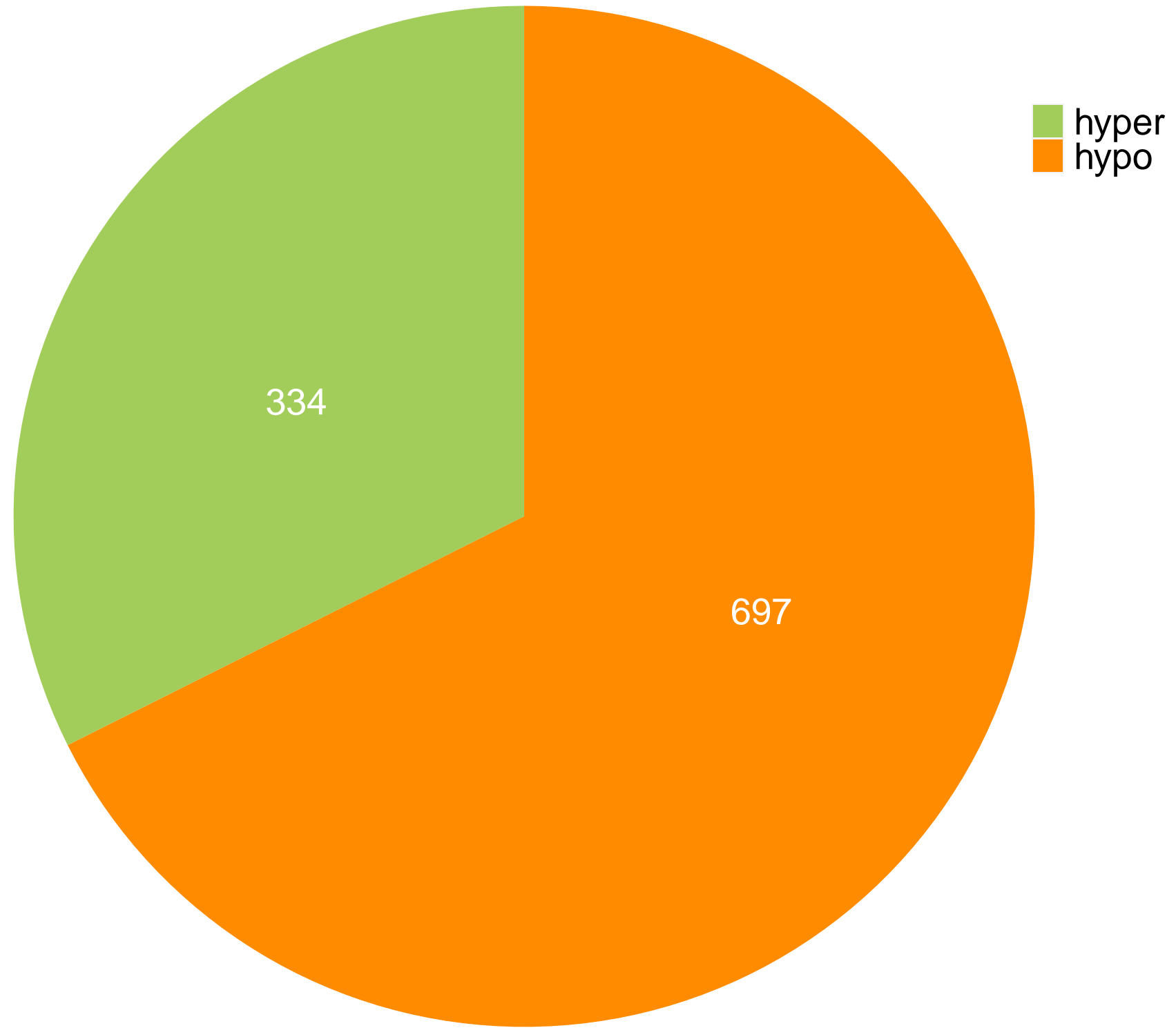

Supplement: Supplementary Materials — Table S1. It included subject information. Table S2. Diagnostic criteria of blood heat syndrome (BHS) and blood stasis syndrome (BSS). Table S3. TREND statement checklist. Figure S1. Sample quality control chart note. The horizontal axis is log2 (methylated median value) and the vertical axis is nonmethylated median value. The overall methylation degree of each sample is represented by a dot, and its distribution in the upper right corner of the dotted line indicated that the quality control standard had been met. All samples in this study met the standard of quality control. Figure S2. Characterization of DMPs in psoriasis vs. normal (N = 875). (a) Orange and green represent the proportion of hypermethylated and hypomethylated DMPs, respectively. (b) Distribution of DMPs in different regions of the genome. (c) Distribution of DMPs in the genome and CpG island regions. (d) Distribution of methylation levels in different regions of genome and CpG islands. Figure S3. Characterization of DMPs in psoriatic BHS (N = 1031) and BSS (N = 1094) vs. normal. (a, b) Orange and green represent the proportion of hypermethylated and hypomethylated DMPs of BHS vs. normal (left) and BSS vs. normal (right), respectively. (c) DMPs in different regions of the genome in psoriasis with BHS. (d) Distribution of DMPs in different regions of the genome in psoriasis patients with BSS. (e) Distribution of DMPs in different regions of CpG islands in psoriasis patients with BHS. (f) Distribution of DMPs in different regions of CpG islands in psoriasis patients with BSS. (g, h) Distribution of methylation levels in different regions of genome and CpG islands. Figure S4. Characterization of DMPs in psoriatic BHS vs. psoriatic BSS (N = 247). (a) Orange and green represent the proportion of hypermethylated and hypomethylated DMPs, respectively. (b) Distribution of DMPs in different regions of the genome. (c) Distribution of DMPs in different regions of CpG islands. (d) Distribution of methylation l [file 9343285.f1.zip › 9343285.f1/Figure S3A.pdf]

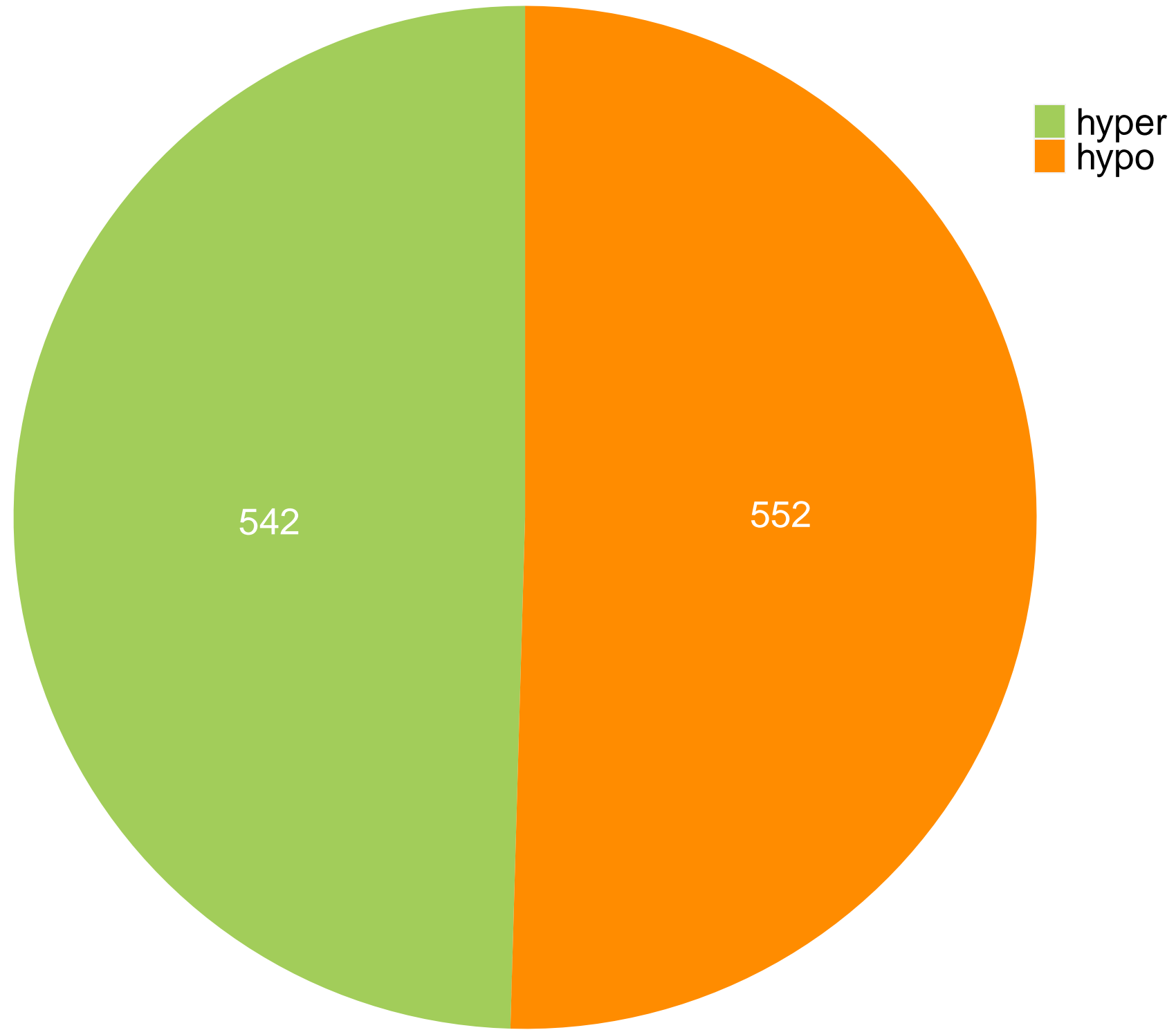

Supplement: Supplementary Materials — Table S1. It included subject information. Table S2. Diagnostic criteria of blood heat syndrome (BHS) and blood stasis syndrome (BSS). Table S3. TREND statement checklist. Figure S1. Sample quality control chart note. The horizontal axis is log2 (methylated median value) and the vertical axis is nonmethylated median value. The overall methylation degree of each sample is represented by a dot, and its distribution in the upper right corner of the dotted line indicated that the quality control standard had been met. All samples in this study met the standard of quality control. Figure S2. Characterization of DMPs in psoriasis vs. normal (N = 875). (a) Orange and green represent the proportion of hypermethylated and hypomethylated DMPs, respectively. (b) Distribution of DMPs in different regions of the genome. (c) Distribution of DMPs in the genome and CpG island regions. (d) Distribution of methylation levels in different regions of genome and CpG islands. Figure S3. Characterization of DMPs in psoriatic BHS (N = 1031) and BSS (N = 1094) vs. normal. (a, b) Orange and green represent the proportion of hypermethylated and hypomethylated DMPs of BHS vs. normal (left) and BSS vs. normal (right), respectively. (c) DMPs in different regions of the genome in psoriasis with BHS. (d) Distribution of DMPs in different regions of the genome in psoriasis patients with BSS. (e) Distribution of DMPs in different regions of CpG islands in psoriasis patients with BHS. (f) Distribution of DMPs in different regions of CpG islands in psoriasis patients with BSS. (g, h) Distribution of methylation levels in different regions of genome and CpG islands. Figure S4. Characterization of DMPs in psoriatic BHS vs. psoriatic BSS (N = 247). (a) Orange and green represent the proportion of hypermethylated and hypomethylated DMPs, respectively. (b) Distribution of DMPs in different regions of the genome. (c) Distribution of DMPs in different regions of CpG islands. (d) Distribution of methylation l [file 9343285.f1.zip › 9343285.f1/Figure S3B.pdf]

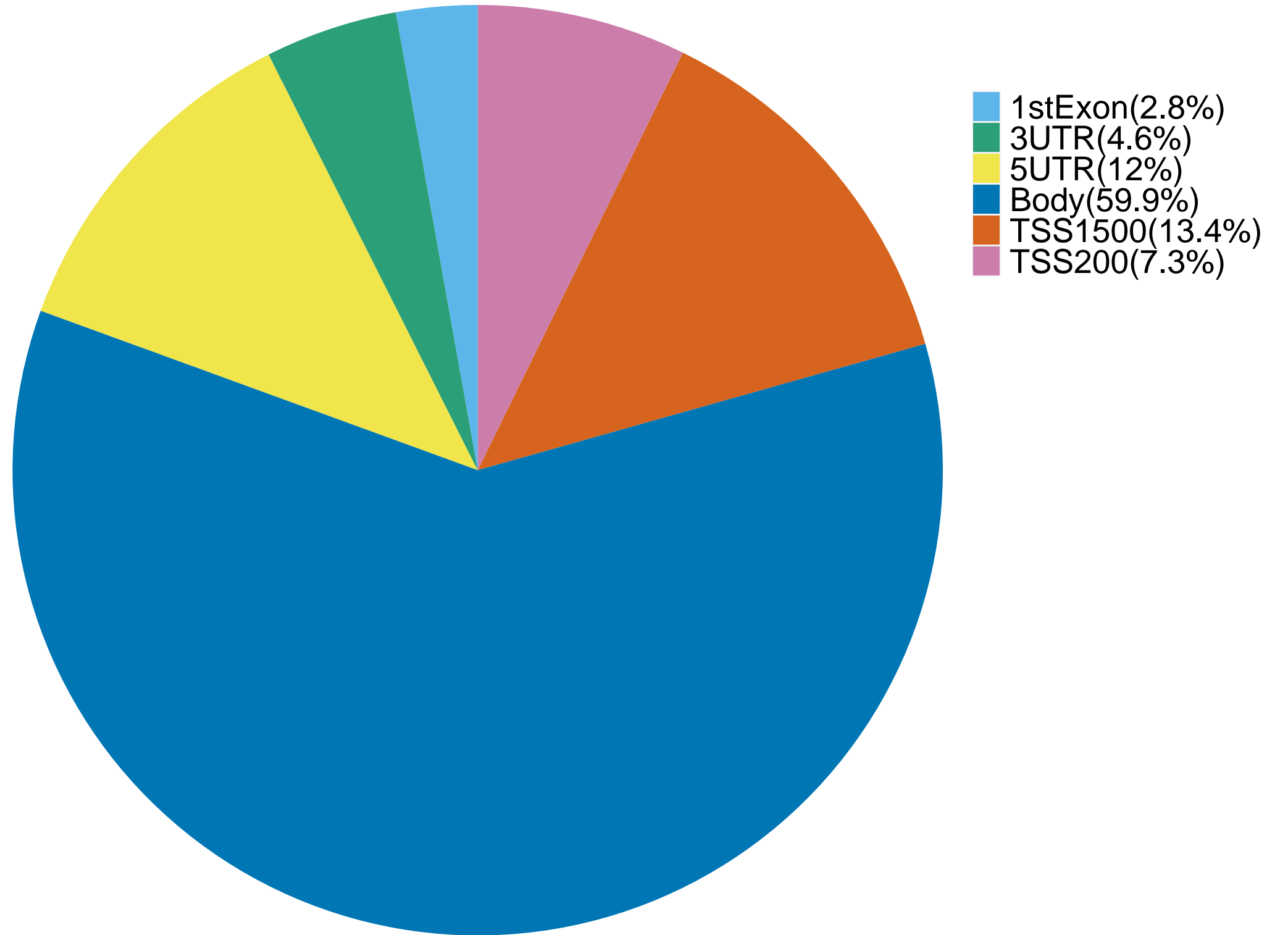

Supplement: Supplementary Materials — Table S1. It included subject information. Table S2. Diagnostic criteria of blood heat syndrome (BHS) and blood stasis syndrome (BSS). Table S3. TREND statement checklist. Figure S1. Sample quality control chart note. The horizontal axis is log2 (methylated median value) and the vertical axis is nonmethylated median value. The overall methylation degree of each sample is represented by a dot, and its distribution in the upper right corner of the dotted line indicated that the quality control standard had been met. All samples in this study met the standard of quality control. Figure S2. Characterization of DMPs in psoriasis vs. normal (N = 875). (a) Orange and green represent the proportion of hypermethylated and hypomethylated DMPs, respectively. (b) Distribution of DMPs in different regions of the genome. (c) Distribution of DMPs in the genome and CpG island regions. (d) Distribution of methylation levels in different regions of genome and CpG islands. Figure S3. Characterization of DMPs in psoriatic BHS (N = 1031) and BSS (N = 1094) vs. normal. (a, b) Orange and green represent the proportion of hypermethylated and hypomethylated DMPs of BHS vs. normal (left) and BSS vs. normal (right), respectively. (c) DMPs in different regions of the genome in psoriasis with BHS. (d) Distribution of DMPs in different regions of the genome in psoriasis patients with BSS. (e) Distribution of DMPs in different regions of CpG islands in psoriasis patients with BHS. (f) Distribution of DMPs in different regions of CpG islands in psoriasis patients with BSS. (g, h) Distribution of methylation levels in different regions of genome and CpG islands. Figure S4. Characterization of DMPs in psoriatic BHS vs. psoriatic BSS (N = 247). (a) Orange and green represent the proportion of hypermethylated and hypomethylated DMPs, respectively. (b) Distribution of DMPs in different regions of the genome. (c) Distribution of DMPs in different regions of CpG islands. (d) Distribution of methylation l [file 9343285.f1.zip › 9343285.f1/Figure S3C.pdf]

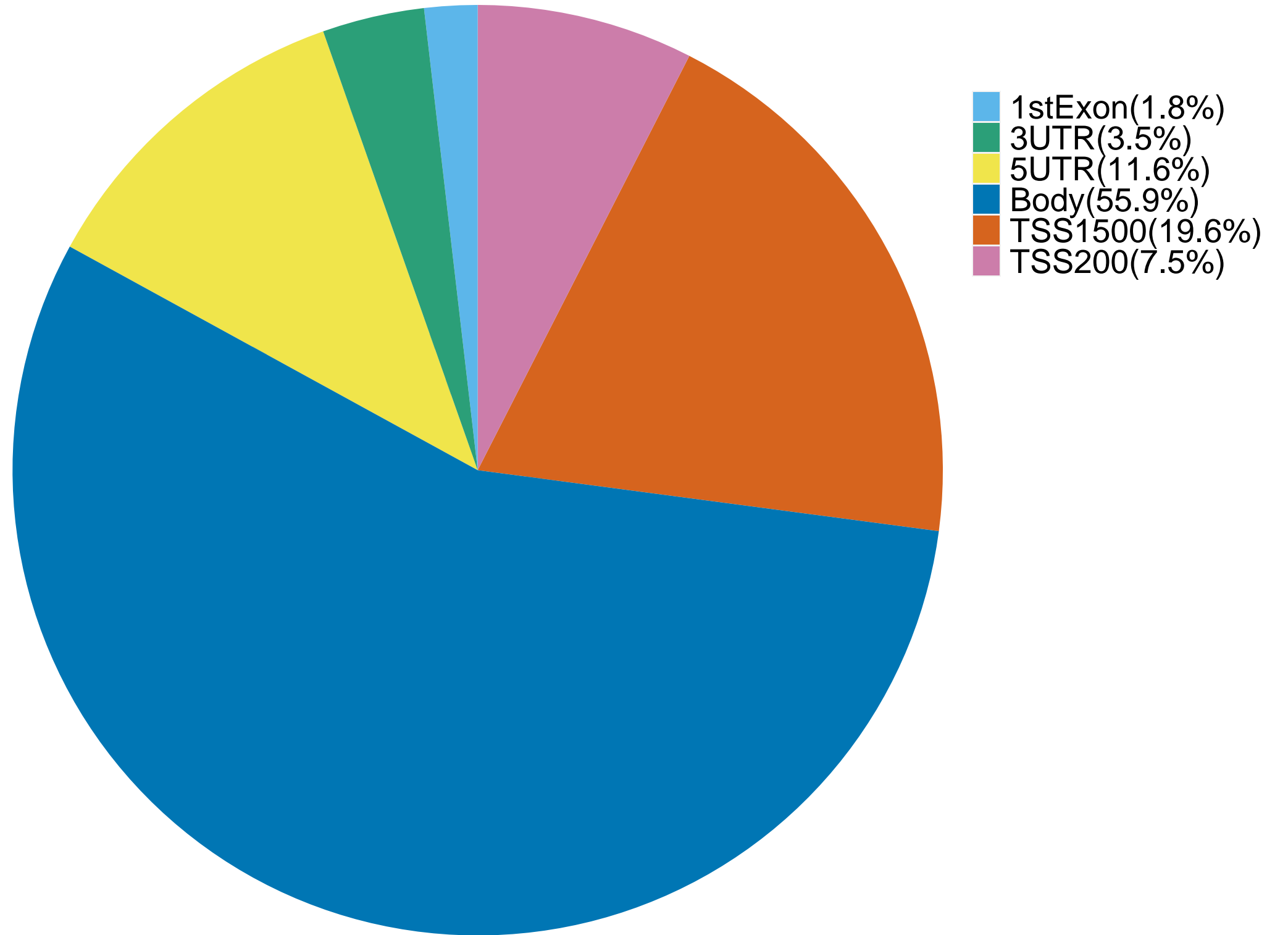

Supplement: Supplementary Materials — Table S1. It included subject information. Table S2. Diagnostic criteria of blood heat syndrome (BHS) and blood stasis syndrome (BSS). Table S3. TREND statement checklist. Figure S1. Sample quality control chart note. The horizontal axis is log2 (methylated median value) and the vertical axis is nonmethylated median value. The overall methylation degree of each sample is represented by a dot, and its distribution in the upper right corner of the dotted line indicated that the quality control standard had been met. All samples in this study met the standard of quality control. Figure S2. Characterization of DMPs in psoriasis vs. normal (N = 875). (a) Orange and green represent the proportion of hypermethylated and hypomethylated DMPs, respectively. (b) Distribution of DMPs in different regions of the genome. (c) Distribution of DMPs in the genome and CpG island regions. (d) Distribution of methylation levels in different regions of genome and CpG islands. Figure S3. Characterization of DMPs in psoriatic BHS (N = 1031) and BSS (N = 1094) vs. normal. (a, b) Orange and green represent the proportion of hypermethylated and hypomethylated DMPs of BHS vs. normal (left) and BSS vs. normal (right), respectively. (c) DMPs in different regions of the genome in psoriasis with BHS. (d) Distribution of DMPs in different regions of the genome in psoriasis patients with BSS. (e) Distribution of DMPs in different regions of CpG islands in psoriasis patients with BHS. (f) Distribution of DMPs in different regions of CpG islands in psoriasis patients with BSS. (g, h) Distribution of methylation levels in different regions of genome and CpG islands. Figure S4. Characterization of DMPs in psoriatic BHS vs. psoriatic BSS (N = 247). (a) Orange and green represent the proportion of hypermethylated and hypomethylated DMPs, respectively. (b) Distribution of DMPs in different regions of the genome. (c) Distribution of DMPs in different regions of CpG islands. (d) Distribution of methylation l [file 9343285.f1.zip › 9343285.f1/Figure S3D.pdf]

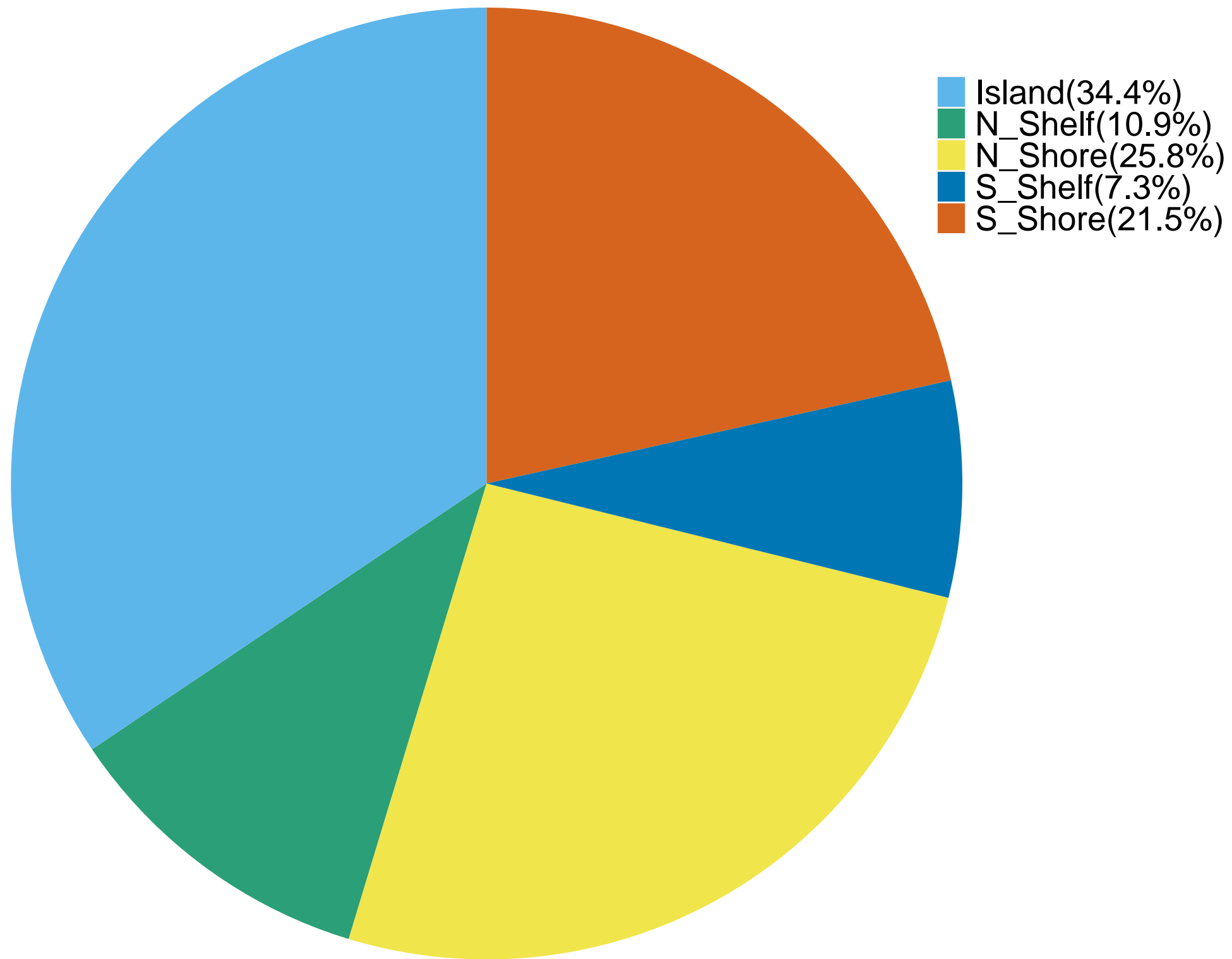

Supplement: Supplementary Materials — Table S1. It included subject information. Table S2. Diagnostic criteria of blood heat syndrome (BHS) and blood stasis syndrome (BSS). Table S3. TREND statement checklist. Figure S1. Sample quality control chart note. The horizontal axis is log2 (methylated median value) and the vertical axis is nonmethylated median value. The overall methylation degree of each sample is represented by a dot, and its distribution in the upper right corner of the dotted line indicated that the quality control standard had been met. All samples in this study met the standard of quality control. Figure S2. Characterization of DMPs in psoriasis vs. normal (N = 875). (a) Orange and green represent the proportion of hypermethylated and hypomethylated DMPs, respectively. (b) Distribution of DMPs in different regions of the genome. (c) Distribution of DMPs in the genome and CpG island regions. (d) Distribution of methylation levels in different regions of genome and CpG islands. Figure S3. Characterization of DMPs in psoriatic BHS (N = 1031) and BSS (N = 1094) vs. normal. (a, b) Orange and green represent the proportion of hypermethylated and hypomethylated DMPs of BHS vs. normal (left) and BSS vs. normal (right), respectively. (c) DMPs in different regions of the genome in psoriasis with BHS. (d) Distribution of DMPs in different regions of the genome in psoriasis patients with BSS. (e) Distribution of DMPs in different regions of CpG islands in psoriasis patients with BHS. (f) Distribution of DMPs in different regions of CpG islands in psoriasis patients with BSS. (g, h) Distribution of methylation levels in different regions of genome and CpG islands. Figure S4. Characterization of DMPs in psoriatic BHS vs. psoriatic BSS (N = 247). (a) Orange and green represent the proportion of hypermethylated and hypomethylated DMPs, respectively. (b) Distribution of DMPs in different regions of the genome. (c) Distribution of DMPs in different regions of CpG islands. (d) Distribution of methylation l [file 9343285.f1.zip › 9343285.f1/Figure S3E.pdf]

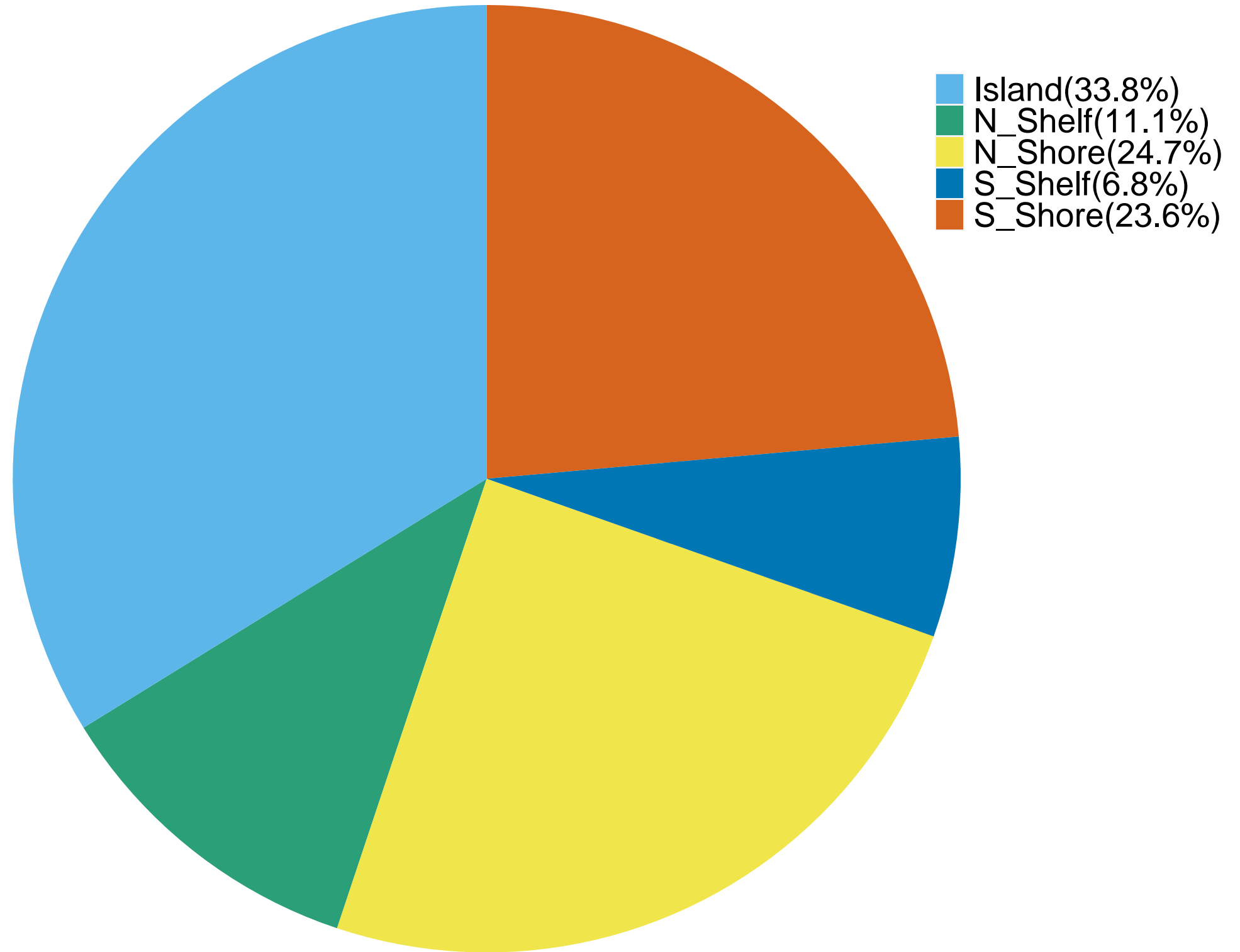

Supplement: Supplementary Materials — Table S1. It included subject information. Table S2. Diagnostic criteria of blood heat syndrome (BHS) and blood stasis syndrome (BSS). Table S3. TREND statement checklist. Figure S1. Sample quality control chart note. The horizontal axis is log2 (methylated median value) and the vertical axis is nonmethylated median value. The overall methylation degree of each sample is represented by a dot, and its distribution in the upper right corner of the dotted line indicated that the quality control standard had been met. All samples in this study met the standard of quality control. Figure S2. Characterization of DMPs in psoriasis vs. normal (N = 875). (a) Orange and green represent the proportion of hypermethylated and hypomethylated DMPs, respectively. (b) Distribution of DMPs in different regions of the genome. (c) Distribution of DMPs in the genome and CpG island regions. (d) Distribution of methylation levels in different regions of genome and CpG islands. Figure S3. Characterization of DMPs in psoriatic BHS (N = 1031) and BSS (N = 1094) vs. normal. (a, b) Orange and green represent the proportion of hypermethylated and hypomethylated DMPs of BHS vs. normal (left) and BSS vs. normal (right), respectively. (c) DMPs in different regions of the genome in psoriasis with BHS. (d) Distribution of DMPs in different regions of the genome in psoriasis patients with BSS. (e) Distribution of DMPs in different regions of CpG islands in psoriasis patients with BHS. (f) Distribution of DMPs in different regions of CpG islands in psoriasis patients with BSS. (g, h) Distribution of methylation levels in different regions of genome and CpG islands. Figure S4. Characterization of DMPs in psoriatic BHS vs. psoriatic BSS (N = 247). (a) Orange and green represent the proportion of hypermethylated and hypomethylated DMPs, respectively. (b) Distribution of DMPs in different regions of the genome. (c) Distribution of DMPs in different regions of CpG islands. (d) Distribution of methylation l [file 9343285.f1.zip › 9343285.f1/Figure S3F.pdf]

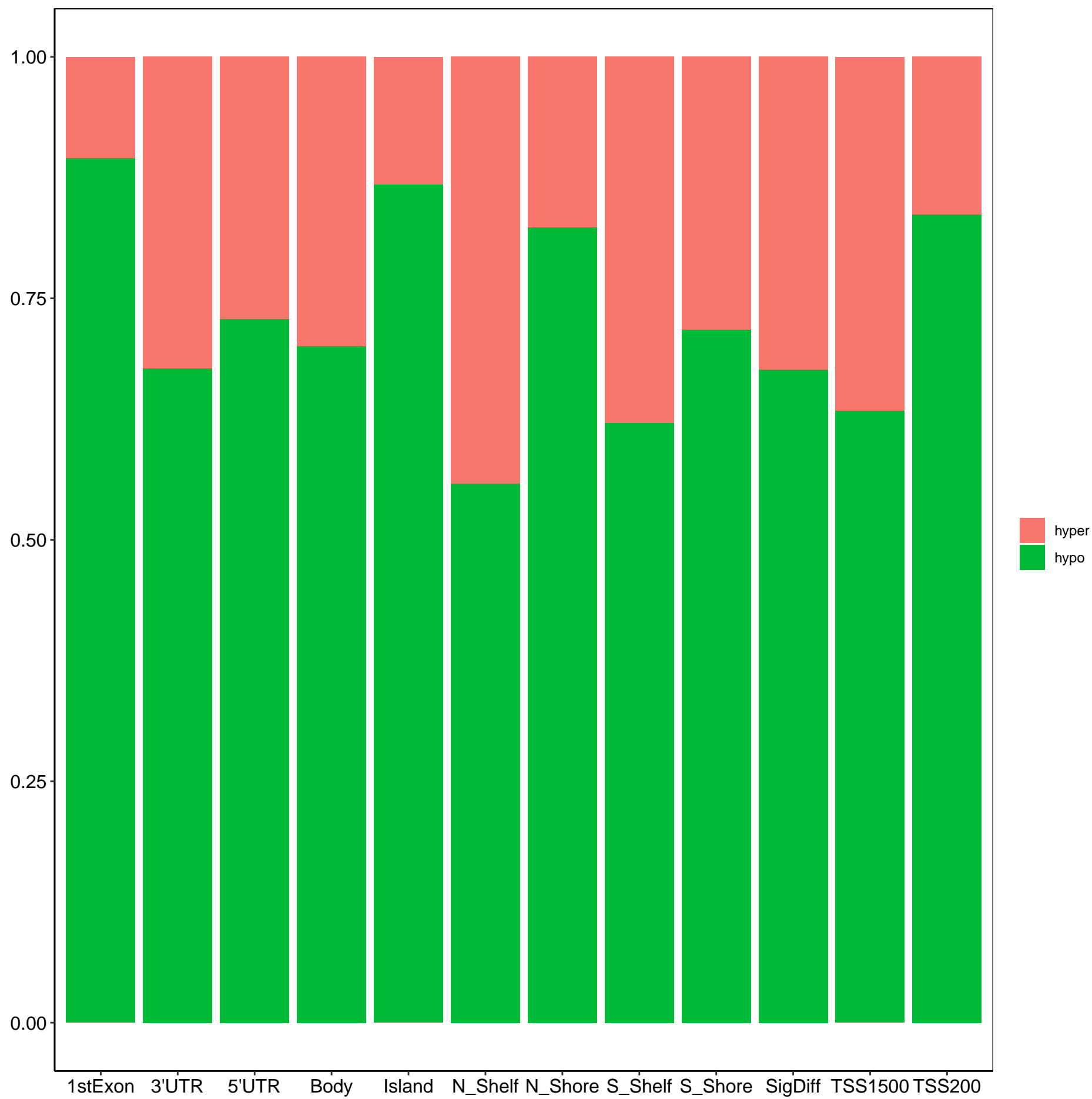

Supplement: Supplementary Materials — Table S1. It included subject information. Table S2. Diagnostic criteria of blood heat syndrome (BHS) and blood stasis syndrome (BSS). Table S3. TREND statement checklist. Figure S1. Sample quality control chart note. The horizontal axis is log2 (methylated median value) and the vertical axis is nonmethylated median value. The overall methylation degree of each sample is represented by a dot, and its distribution in the upper right corner of the dotted line indicated that the quality control standard had been met. All samples in this study met the standard of quality control. Figure S2. Characterization of DMPs in psoriasis vs. normal (N = 875). (a) Orange and green represent the proportion of hypermethylated and hypomethylated DMPs, respectively. (b) Distribution of DMPs in different regions of the genome. (c) Distribution of DMPs in the genome and CpG island regions. (d) Distribution of methylation levels in different regions of genome and CpG islands. Figure S3. Characterization of DMPs in psoriatic BHS (N = 1031) and BSS (N = 1094) vs. normal. (a, b) Orange and green represent the proportion of hypermethylated and hypomethylated DMPs of BHS vs. normal (left) and BSS vs. normal (right), respectively. (c) DMPs in different regions of the genome in psoriasis with BHS. (d) Distribution of DMPs in different regions of the genome in psoriasis patients with BSS. (e) Distribution of DMPs in different regions of CpG islands in psoriasis patients with BHS. (f) Distribution of DMPs in different regions of CpG islands in psoriasis patients with BSS. (g, h) Distribution of methylation levels in different regions of genome and CpG islands. Figure S4. Characterization of DMPs in psoriatic BHS vs. psoriatic BSS (N = 247). (a) Orange and green represent the proportion of hypermethylated and hypomethylated DMPs, respectively. (b) Distribution of DMPs in different regions of the genome. (c) Distribution of DMPs in different regions of CpG islands. (d) Distribution of methylation l [file 9343285.f1.zip › 9343285.f1/Figure S3G.pdf]

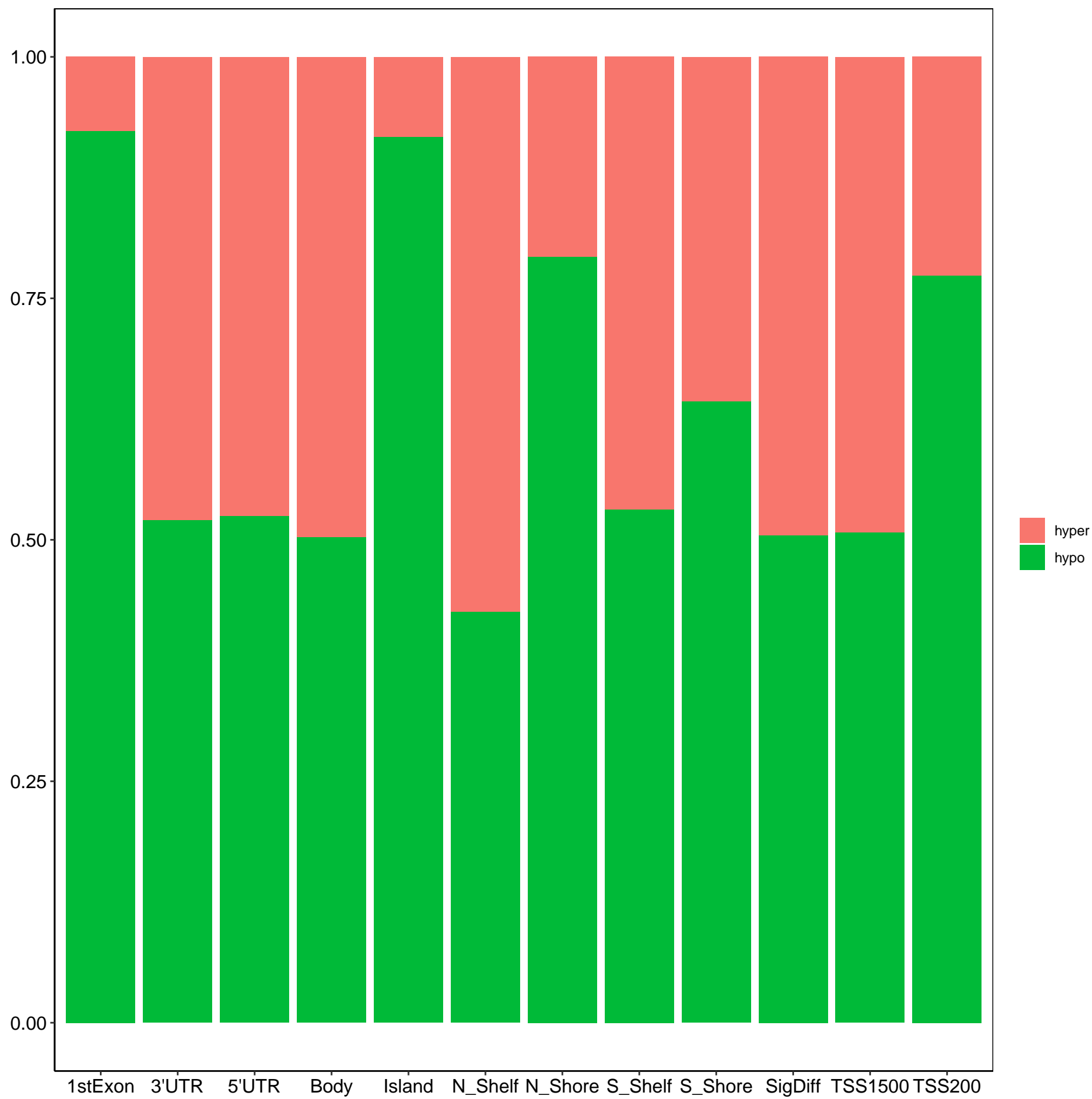

Supplement: Supplementary Materials — Table S1. It included subject information. Table S2. Diagnostic criteria of blood heat syndrome (BHS) and blood stasis syndrome (BSS). Table S3. TREND statement checklist. Figure S1. Sample quality control chart note. The horizontal axis is log2 (methylated median value) and the vertical axis is nonmethylated median value. The overall methylation degree of each sample is represented by a dot, and its distribution in the upper right corner of the dotted line indicated that the quality control standard had been met. All samples in this study met the standard of quality control. Figure S2. Characterization of DMPs in psoriasis vs. normal (N = 875). (a) Orange and green represent the proportion of hypermethylated and hypomethylated DMPs, respectively. (b) Distribution of DMPs in different regions of the genome. (c) Distribution of DMPs in the genome and CpG island regions. (d) Distribution of methylation levels in different regions of genome and CpG islands. Figure S3. Characterization of DMPs in psoriatic BHS (N = 1031) and BSS (N = 1094) vs. normal. (a, b) Orange and green represent the proportion of hypermethylated and hypomethylated DMPs of BHS vs. normal (left) and BSS vs. normal (right), respectively. (c) DMPs in different regions of the genome in psoriasis with BHS. (d) Distribution of DMPs in different regions of the genome in psoriasis patients with BSS. (e) Distribution of DMPs in different regions of CpG islands in psoriasis patients with BHS. (f) Distribution of DMPs in different regions of CpG islands in psoriasis patients with BSS. (g, h) Distribution of methylation levels in different regions of genome and CpG islands. Figure S4. Characterization of DMPs in psoriatic BHS vs. psoriatic BSS (N = 247). (a) Orange and green represent the proportion of hypermethylated and hypomethylated DMPs, respectively. (b) Distribution of DMPs in different regions of the genome. (c) Distribution of DMPs in different regions of CpG islands. (d) Distribution of methylation l [file 9343285.f1.zip › 9343285.f1/Figure S3H.pdf]

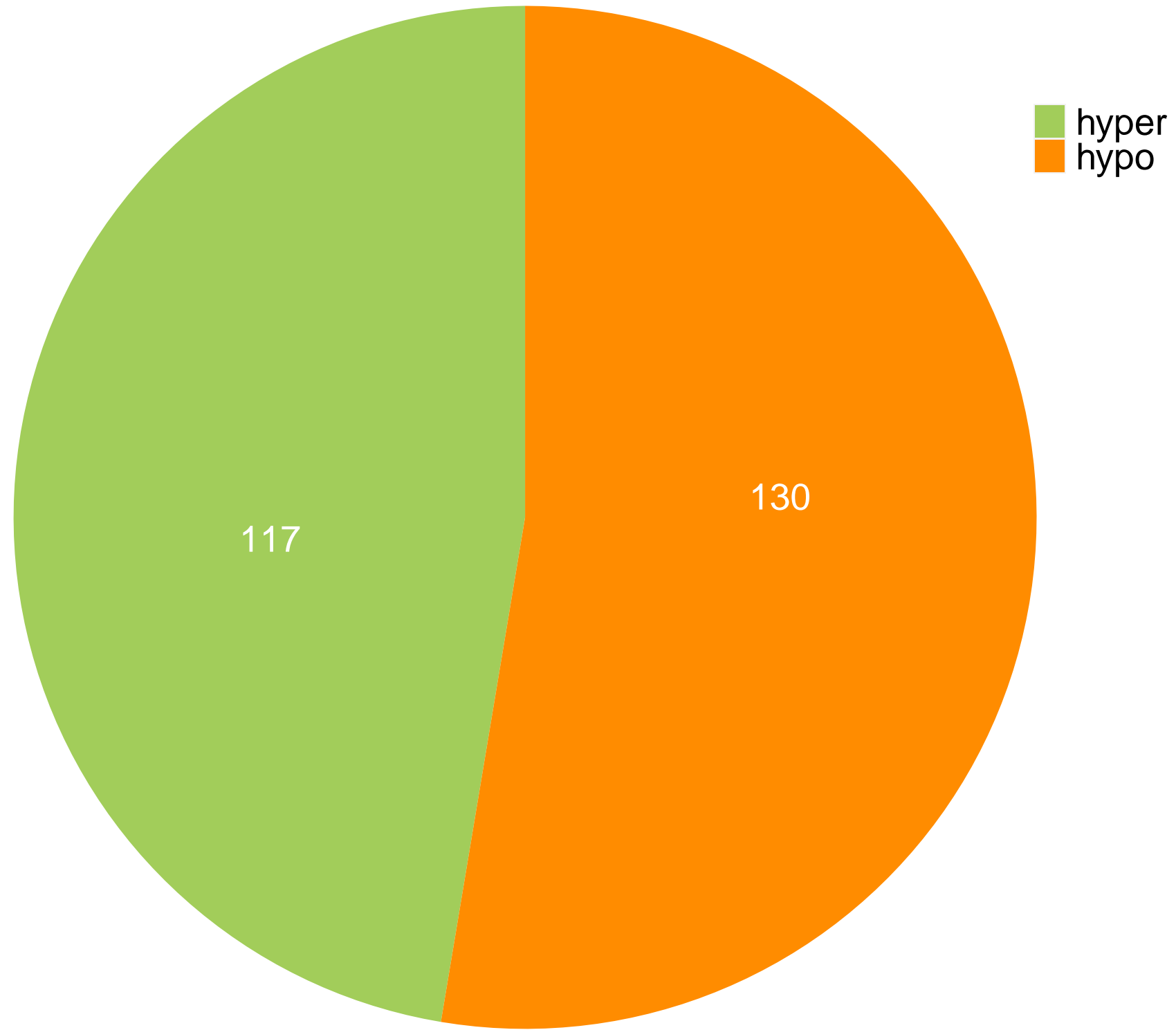

Supplement: Supplementary Materials — Table S1. It included subject information. Table S2. Diagnostic criteria of blood heat syndrome (BHS) and blood stasis syndrome (BSS). Table S3. TREND statement checklist. Figure S1. Sample quality control chart note. The horizontal axis is log2 (methylated median value) and the vertical axis is nonmethylated median value. The overall methylation degree of each sample is represented by a dot, and its distribution in the upper right corner of the dotted line indicated that the quality control standard had been met. All samples in this study met the standard of quality control. Figure S2. Characterization of DMPs in psoriasis vs. normal (N = 875). (a) Orange and green represent the proportion of hypermethylated and hypomethylated DMPs, respectively. (b) Distribution of DMPs in different regions of the genome. (c) Distribution of DMPs in the genome and CpG island regions. (d) Distribution of methylation levels in different regions of genome and CpG islands. Figure S3. Characterization of DMPs in psoriatic BHS (N = 1031) and BSS (N = 1094) vs. normal. (a, b) Orange and green represent the proportion of hypermethylated and hypomethylated DMPs of BHS vs. normal (left) and BSS vs. normal (right), respectively. (c) DMPs in different regions of the genome in psoriasis with BHS. (d) Distribution of DMPs in different regions of the genome in psoriasis patients with BSS. (e) Distribution of DMPs in different regions of CpG islands in psoriasis patients with BHS. (f) Distribution of DMPs in different regions of CpG islands in psoriasis patients with BSS. (g, h) Distribution of methylation levels in different regions of genome and CpG islands. Figure S4. Characterization of DMPs in psoriatic BHS vs. psoriatic BSS (N = 247). (a) Orange and green represent the proportion of hypermethylated and hypomethylated DMPs, respectively. (b) Distribution of DMPs in different regions of the genome. (c) Distribution of DMPs in different regions of CpG islands. (d) Distribution of methylation l [file 9343285.f1.zip › 9343285.f1/Figure S4A.pdf]

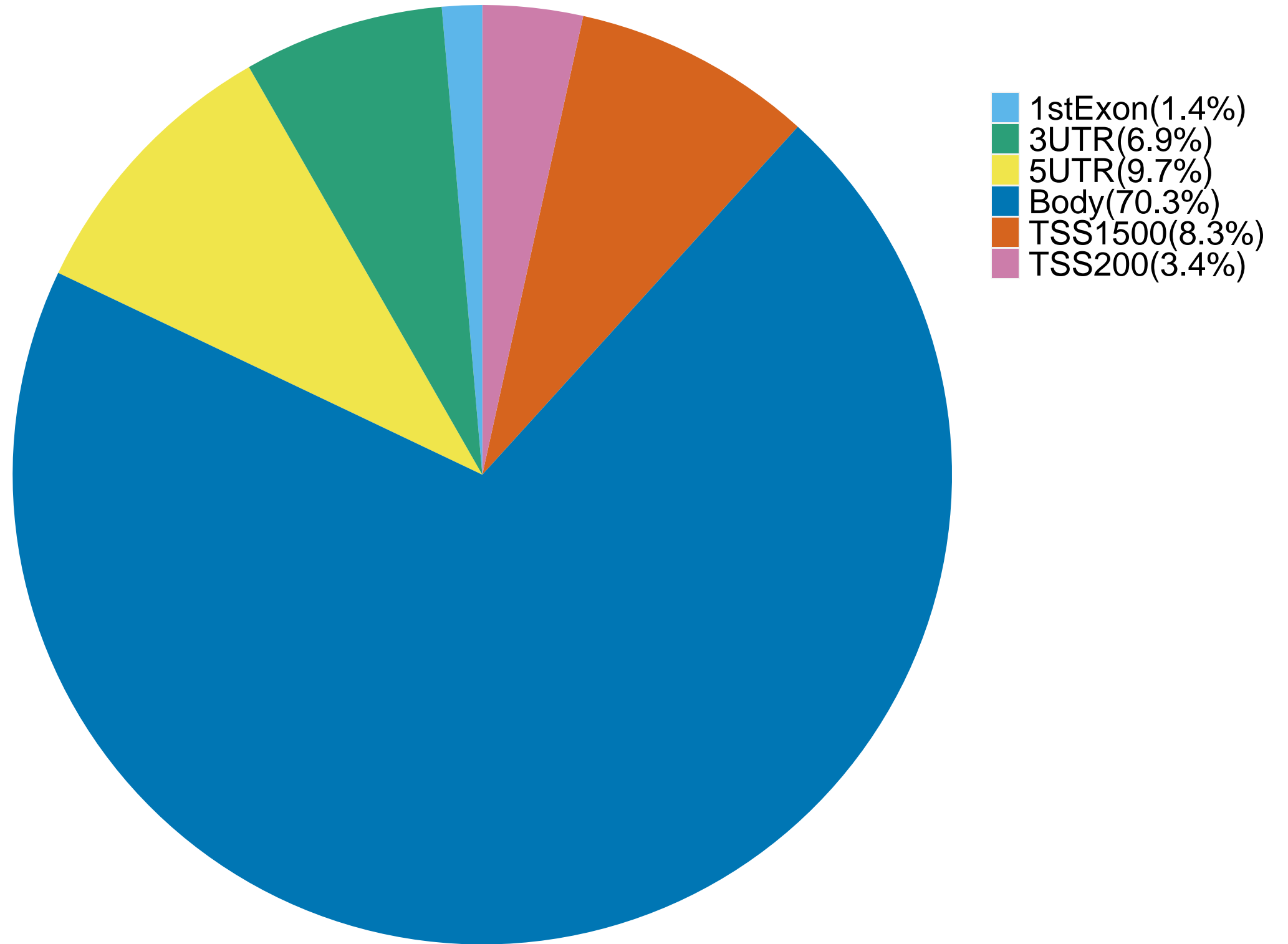

Supplement: Supplementary Materials — Table S1. It included subject information. Table S2. Diagnostic criteria of blood heat syndrome (BHS) and blood stasis syndrome (BSS). Table S3. TREND statement checklist. Figure S1. Sample quality control chart note. The horizontal axis is log2 (methylated median value) and the vertical axis is nonmethylated median value. The overall methylation degree of each sample is represented by a dot, and its distribution in the upper right corner of the dotted line indicated that the quality control standard had been met. All samples in this study met the standard of quality control. Figure S2. Characterization of DMPs in psoriasis vs. normal (N = 875). (a) Orange and green represent the proportion of hypermethylated and hypomethylated DMPs, respectively. (b) Distribution of DMPs in different regions of the genome. (c) Distribution of DMPs in the genome and CpG island regions. (d) Distribution of methylation levels in different regions of genome and CpG islands. Figure S3. Characterization of DMPs in psoriatic BHS (N = 1031) and BSS (N = 1094) vs. normal. (a, b) Orange and green represent the proportion of hypermethylated and hypomethylated DMPs of BHS vs. normal (left) and BSS vs. normal (right), respectively. (c) DMPs in different regions of the genome in psoriasis with BHS. (d) Distribution of DMPs in different regions of the genome in psoriasis patients with BSS. (e) Distribution of DMPs in different regions of CpG islands in psoriasis patients with BHS. (f) Distribution of DMPs in different regions of CpG islands in psoriasis patients with BSS. (g, h) Distribution of methylation levels in different regions of genome and CpG islands. Figure S4. Characterization of DMPs in psoriatic BHS vs. psoriatic BSS (N = 247). (a) Orange and green represent the proportion of hypermethylated and hypomethylated DMPs, respectively. (b) Distribution of DMPs in different regions of the genome. (c) Distribution of DMPs in different regions of CpG islands. (d) Distribution of methylation l [file 9343285.f1.zip › 9343285.f1/Figure S4B.pdf]

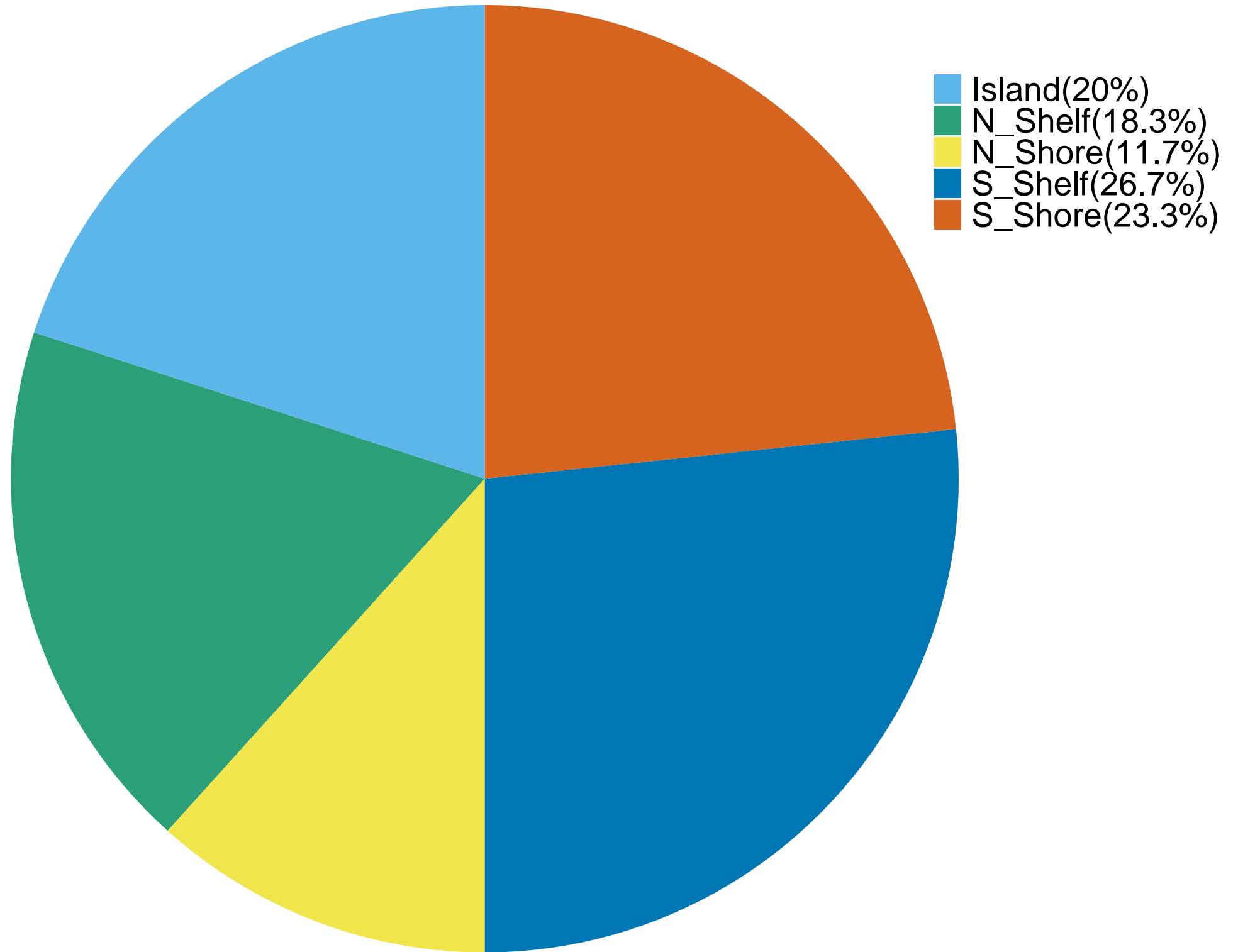

Supplement: Supplementary Materials — Table S1. It included subject information. Table S2. Diagnostic criteria of blood heat syndrome (BHS) and blood stasis syndrome (BSS). Table S3. TREND statement checklist. Figure S1. Sample quality control chart note. The horizontal axis is log2 (methylated median value) and the vertical axis is nonmethylated median value. The overall methylation degree of each sample is represented by a dot, and its distribution in the upper right corner of the dotted line indicated that the quality control standard had been met. All samples in this study met the standard of quality control. Figure S2. Characterization of DMPs in psoriasis vs. normal (N = 875). (a) Orange and green represent the proportion of hypermethylated and hypomethylated DMPs, respectively. (b) Distribution of DMPs in different regions of the genome. (c) Distribution of DMPs in the genome and CpG island regions. (d) Distribution of methylation levels in different regions of genome and CpG islands. Figure S3. Characterization of DMPs in psoriatic BHS (N = 1031) and BSS (N = 1094) vs. normal. (a, b) Orange and green represent the proportion of hypermethylated and hypomethylated DMPs of BHS vs. normal (left) and BSS vs. normal (right), respectively. (c) DMPs in different regions of the genome in psoriasis with BHS. (d) Distribution of DMPs in different regions of the genome in psoriasis patients with BSS. (e) Distribution of DMPs in different regions of CpG islands in psoriasis patients with BHS. (f) Distribution of DMPs in different regions of CpG islands in psoriasis patients with BSS. (g, h) Distribution of methylation levels in different regions of genome and CpG islands. Figure S4. Characterization of DMPs in psoriatic BHS vs. psoriatic BSS (N = 247). (a) Orange and green represent the proportion of hypermethylated and hypomethylated DMPs, respectively. (b) Distribution of DMPs in different regions of the genome. (c) Distribution of DMPs in different regions of CpG islands. (d) Distribution of methylation l [file 9343285.f1.zip › 9343285.f1/Figure S4C.pdf]

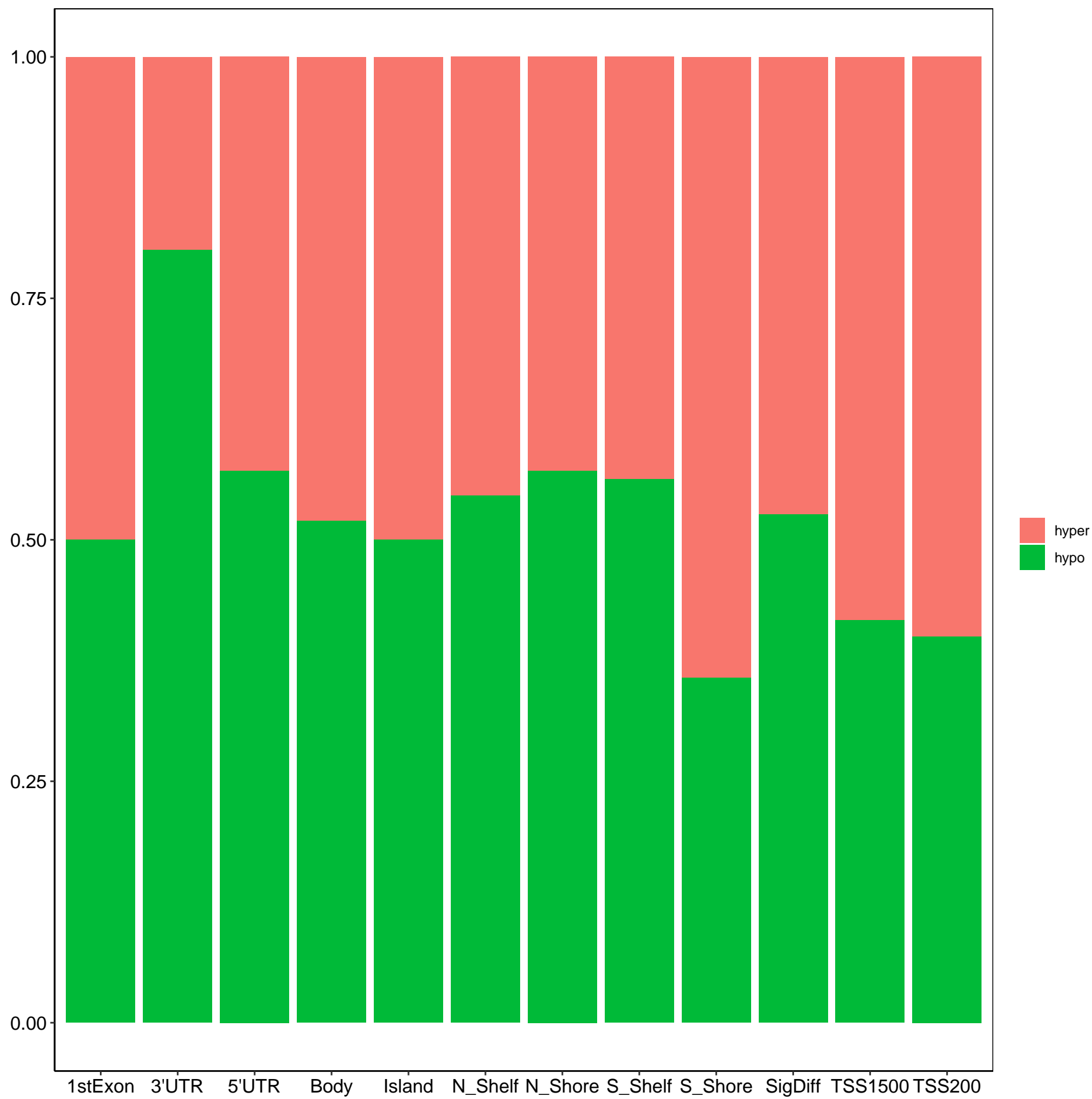

Supplement: Supplementary Materials — Table S1. It included subject information. Table S2. Diagnostic criteria of blood heat syndrome (BHS) and blood stasis syndrome (BSS). Table S3. TREND statement checklist. Figure S1. Sample quality control chart note. The horizontal axis is log2 (methylated median value) and the vertical axis is nonmethylated median value. The overall methylation degree of each sample is represented by a dot, and its distribution in the upper right corner of the dotted line indicated that the quality control standard had been met. All samples in this study met the standard of quality control. Figure S2. Characterization of DMPs in psoriasis vs. normal (N = 875). (a) Orange and green represent the proportion of hypermethylated and hypomethylated DMPs, respectively. (b) Distribution of DMPs in different regions of the genome. (c) Distribution of DMPs in the genome and CpG island regions. (d) Distribution of methylation levels in different regions of genome and CpG islands. Figure S3. Characterization of DMPs in psoriatic BHS (N = 1031) and BSS (N = 1094) vs. normal. (a, b) Orange and green represent the proportion of hypermethylated and hypomethylated DMPs of BHS vs. normal (left) and BSS vs. normal (right), respectively. (c) DMPs in different regions of the genome in psoriasis with BHS. (d) Distribution of DMPs in different regions of the genome in psoriasis patients with BSS. (e) Distribution of DMPs in different regions of CpG islands in psoriasis patients with BHS. (f) Distribution of DMPs in different regions of CpG islands in psoriasis patients with BSS. (g, h) Distribution of methylation levels in different regions of genome and CpG islands. Figure S4. Characterization of DMPs in psoriatic BHS vs. psoriatic BSS (N = 247). (a) Orange and green represent the proportion of hypermethylated and hypomethylated DMPs, respectively. (b) Distribution of DMPs in different regions of the genome. (c) Distribution of DMPs in different regions of CpG islands. (d) Distribution of methylation l [file 9343285.f1.zip › 9343285.f1/Figure S4D.pdf]

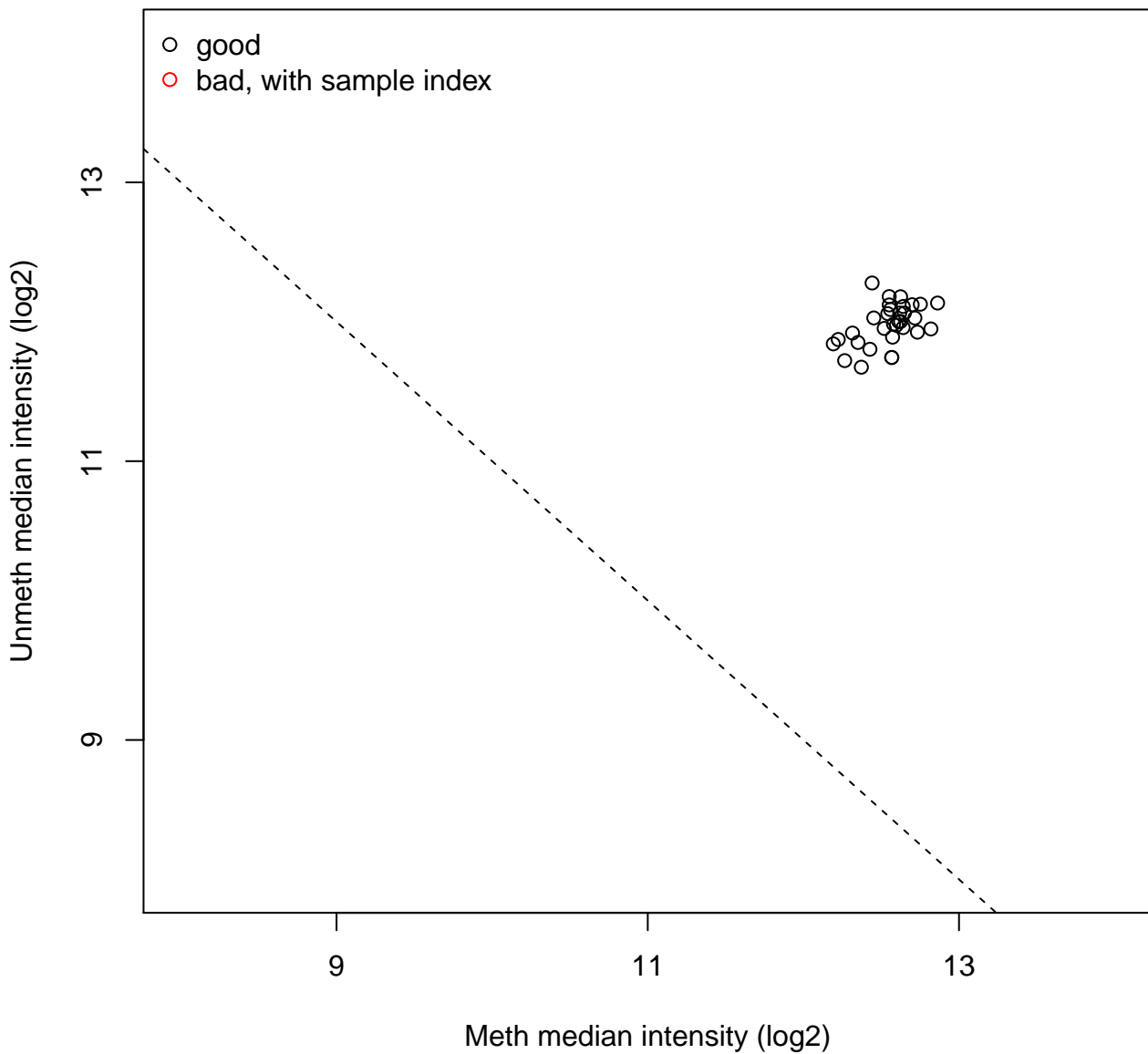

Supplement: Supplementary Materials — Table S1. It included subject information. Table S2. Diagnostic criteria of blood heat syndrome (BHS) and blood stasis syndrome (BSS). Table S3. TREND statement checklist. Figure S1. Sample quality control chart note. The horizontal axis is log2 (methylated median value) and the vertical axis is nonmethylated median value. The overall methylation degree of each sample is represented by a dot, and its distribution in the upper right corner of the dotted line indicated that the quality control standard had been met. All samples in this study met the standard of quality control. Figure S2. Characterization of DMPs in psoriasis vs. normal (N = 875). (a) Orange and green represent the proportion of hypermethylated and hypomethylated DMPs, respectively. (b) Distribution of DMPs in different regions of the genome. (c) Distribution of DMPs in the genome and CpG island regions. (d) Distribution of methylation levels in different regions of genome and CpG islands. Figure S3. Characterization of DMPs in psoriatic BHS (N = 1031) and BSS (N = 1094) vs. normal. (a, b) Orange and green represent the proportion of hypermethylated and hypomethylated DMPs of BHS vs. normal (left) and BSS vs. normal (right), respectively. (c) DMPs in different regions of the genome in psoriasis with BHS. (d) Distribution of DMPs in different regions of the genome in psoriasis patients with BSS. (e) Distribution of DMPs in different regions of CpG islands in psoriasis patients with BHS. (f) Distribution of DMPs in different regions of CpG islands in psoriasis patients with BSS. (g, h) Distribution of methylation levels in different regions of genome and CpG islands. Figure S4. Characterization of DMPs in psoriatic BHS vs. psoriatic BSS (N = 247). (a) Orange and green represent the proportion of hypermethylated and hypomethylated DMPs, respectively. (b) Distribution of DMPs in different regions of the genome. (c) Distribution of DMPs in different regions of CpG islands. (d) Distribution of methylation l [file 9343285.f1.zip › 9343285.f1/FigureS1.pdf]
